# Multiplayer Reach–Avoid Differential Games in 3D Space Inspired by Harris’ Hawks’ Cooperative Hunting Tactics

**DOI:** 10.34133/research.0246

**Published:** 2023-11-30

**Authors:** Wanying Ruan, Haibin Duan, Yongbin Sun, Wanmai Yuan, Jie Xia

**Affiliations:** ^1^ State Key Laboratory of Virtual Reality Technology and Systems, School of Automation Science and Electrical Engineering, Beihang University, Beijing, China.; ^2^ Peng Cheng Laboratory, Shenzhen, China.; ^3^ Information Science Academy of CETC, Beijing, China.

## Abstract

This paper investigates a multiplayer reach–avoid differential game in 3-dimensional (3D) space, which involves multiple pursuers, multiple evaders, and a designated target region. The evaders aim to reach the target region, while the pursuers attempt to guard the target region by capturing the evaders. This class of research holds significant practical value. However, the complexity of the problem escalates substantially with the growing number of players, rendering its solution extremely challenging. In this paper, the multiplayer game is divided into many subgames considering the cooperation among pursuers, reducing the computational burden, and obtaining numerically tractable strategies for players. First, the Apollonius sphere, a fundamental geometric tool for analyzing the 3D differential game, is formulated, and its properties are proved. Based on this, the optimal interception point for the pursuer to capture the evader is derived and the winning conditions for the pursuer and evader are established. Then, based on the Apollonius sphere, the optimal state feedback strategies of players are designed, and simultaneously, the optimal one-to-one pairings are obtained. Meanwhile, the Value function of the multiplayer reach–avoid differential game is explicitly given and is proved to satisfy Hamilton–Jacobi–Isaacs (HJI) equation. Moreover, the matching algorithm for the case with pursuers outnumbered evaders is provided through constructing a weighted bipartite graph, and the cooperative tactics for multiple pursuers are proposed, inspired by the Harris’ Hawks intelligent cooperative hunting tactics. Finally, numerical simulations are conducted to illustrate the effectiveness of the theoretical results for both cases where the number of adversary players is equal and unequal between the 2 groups.

## Introduction

Multiplayer reach–avoid differential game involves 2 adversary teams and a target region, and the players are divided into the pursuers team and the evaders team. The objective of the pursuers is to prevent the evader from approaching the target region and capture all evaders, and the evaders aim to reach the target region as much as possible [1]. This problem is derived from the actual civil, industrial, and military backgrounds [2,3], which is motivated by the applications in the field of autonomous robots and aerospace [4–6], such as border defense, missile interception, region security, and unmanned aerial vehicle (UAV) target guarding, which are all its instantiation research problems [7–9].

Reach–avoid differential game is a development based on the classic pursuit–evasion differential game [10], initially proposed in Issacs’ seminal work [11]. Compared with the pursuit–evasion game, the distinguishing characteristic of the reach–avoid game lies in the consideration of the target region, resulting in a more complex game goal with both cooperation and conflict among players. In the pursuit–evasion game, the players only need to consider a single purpose: The pursuer focuses solely on minimizing the distance from the evader to eventually capture it, while the evader aims solely to evade the pursuer. Conversely, in the reach–avoid game, the players need to consider dual purpose: The pursuer, also known as the defender, attempts to protect the target region from the evader while trying to catch the evader. While the evader, also known as the attacker, has to strategize how to reach the target region while avoiding capture. Due to its greater complexity and practical application significance, the reach–avoid game has obtained increased attention from researchers in recent years.

The conventional method for solving the differential game is the numerical solution based on Hamilton–Jacobi–Isaacs (HJI) equation. By solving the HJI partial differential equation, one can determine the winning regions for each player as well as their optimal strategies derived from the gradient of the HJI solution. However, obtaining an analytical solution using the HJI approach is challenging and typically relies on numerical tools such as level sets. In the context of a 2-player capture-the-flag differential game, the HJI method based on the level set is employed [12]. This allows for obtaining a numerically approximated solution to the HJI partial differential equation through numerical interpolations, then the winning regions for 2 players and each player’s winning strategies are derived from the level set. HJI approach is particularly effective for one pursuer one evader differential game but suffers from the curse of dimensionality with the increasing number of players, and the computational complexity increases exponentially, making it almost impossible to solve. A reachable set is also a common method to address differential games. The game of kind can be analyzed through the reachable set, and the winning region of the player can be depicted by the boundary of the reachable set. The cooperative strategies for the target and defender are designed using reachability analysis to cooperate against a faster attacker [13]. Reachability analysis is beneficial for low-dimensional single-objective set problems, but when considering multiplayer and multi-objective sets in the reach–avoid game, it will be very tricky.

As the research on the reach–avoid differential game advances, geometric approaches are favored by researchers because of their excellent properties, among which Voronoi and Apollonius circle are 2 effective geometric tools. A cooperative pursuit algorithm for multiple pursuers against a single evader is presented based on minimizing the area of the generalized Voronoi partition of the evader, and the safe-reachable set is used to tackle the situation of nonconvex domains with unequal speeds [14]. The dominance regions are constructed using the Apollonius circle to provide a complete solution to the P3 (prey, protector, predator) game in the presence of obstacles [15]. A named fishing game is addressed in [16], where a faster evader aims to pass through the gap between 2 pursuers, and the fishing game is divided into included angle subgame and the distance subgame, which is solved by constructing 2 Apollonius circles, and finally the barrier and the optimal control strategies are obtained. Although the Voronoi and the Apollonius circle offer valuable insights, they are limited to scenarios in the plane and cannot be directly applied to solve problems in 3-dimensional (3D) space games. Consequently, this motivates this paper to investigate a geometric analysis tool that can be utilized in 3D space.

However, most existing research has focused on small-scale pursuit–evasion games such as one-to-one and two-to-one scenarios, with few research results on the multiple-to-multiple games, which are still in the exploration stage. The multiplayer target–attacker–defender differential game in the plane with a double integrator is considered in [17], where the optimal control strategies and the attacker-defender matching pairs are derived based on isochrones, to achieve the maximum interception of attackers in the shortest time. Multiplayer reach–avoid game in the plane via pairwise outcomes is proposed in [18], where the multi-pursuer and the multi-evader game is decomposed into the one-on-one case through the graph theoretic maximum matching, and then the HJI partial differential equation is solved for each pair, which effectively reduces the computational complexity. Multiple pursuers multiple evaders differential games in the plane are studied in [19], the optimal assignments and optimal strategies are simultaneously obtained utilizing the Apollonius circle, and the curse of dimensionality is overcome. This paper draws on its ideas and extends it to 3D space.

There have been some studies on differential games in 3D space, albeit with a small number of players. The optimal guidance strategies for the target defense differential game in 3D are addressed in [20], which involves one pursuer, one defender, and a non-maneuverable target, and a pursuit–evasion game in 3D is delineated by setting the geometric relative relationship of the Cartesian space and the players. Two-pursuer one-evader game in 3D space is considered in [21], and the game also degenerates into the one-pursuer one-evader game, and then the barrier and the saddle-point strategies are provided. Similar to this paper, a multiplayer reach–avoid differential game in 3D space is studied in [22], where the maximum bipartite matching is solved by the receding horizon method, and an evasion space is constructed to derive the pursuer winning strategies.

As the above analysis indicates, there has been limited research on multiplayer reach–avoid games in 3D space to date. This paper attempts to conduct exploratory research on this topic and provide valuable insights. Creatures in nature can always bring inspiration to people [23–27]. The optimal evasive strategies of multiple players are designed based on the animals' predator–prey intelligent behavior [28]. The cooperative pursuit strategy of multiple pursuers is proposed, inspired by the swarming behavior of male and female mosquitoes [29]. Harris’ Hawks are a rare social bird of prey, they breed, feed, and hunt in groups, known as the wolf pack of the sky. This paper draws inspiration from the intelligent behavior of Harris’ Hawks in cooperative hunting and proposes cooperative tactics for pursuers combined with differential games to address the multiplayer reach–avoid game in 3D space. The main contributions of this paper are as follows.

a. A geometric approach based on the Apollonius sphere is adopted to address the multiplayer differential game in 3D space in this paper. The geometric properties of the Apollonius sphere are demonstrated, with relevant theorems and corollaries provided for solving differential games in 3D space, which can guide us to solving the game of kind and the game of degree. This geometric approach can be used to tackle any adversarial players with different speeds and serve as a fundamental analytical tool for the differential game in 3D space.

b. The whole game is divided into many subgames involving the one-to-one case and multiple-to-one case. For the one-to-one paired multiplayer differential game, the optimal state feedback strategies are designed for players based on the Apollonius sphere, and simultaneously, the guaranteed pursuers winning strategies and the optimal pairing of one pursuer and one evader are given. The analytic expression of the Value function is provided, which is proved to satisfy the HJI equation.

c. For the multiplayer differential game with multiple-to-one assignments, the cooperative strategy for multiple pursuers against one evader is proposed, simulating the cooperative hunting tactics of Harris’ Hawks. The weighted bipartite graph is constructed according to the optimal interception point, and the matching algorithm of multiple pursuers and multiple evaders is provided.

The rest of this paper is organized as follows. The “Multiplayer Reach–Avoid Differential Game in 3D” section describes the multiplayer reach–avoid differential game in 3D and provides a generic theorem for the differential game. The “Apollonius Sphere” section introduces the properties of the Apollonius sphere and presents the important theorem and corollaries based on the Apollonius sphere used to solve 3D differential games. The “Two Pursuers Two Evaders 3D Reach–Avoid Differential Game” section addresses the 2 pursuers 2 evaders 3D reach–avoid differential game. The “Multiplayer 3D Reach–Avoid Differential Games via Harris’ Hawks Cooperative Hunting Tactics” section presents the optimal strategies and cooperative tactics for the multiplayer reach–avoid differential game in 3D. Simulation results are given in the “Simulation Results” section. Finally, the paper is concluded in the “Conclusions” section.

## Multiplayer Reach–Avoid Differential Game in 3D

### Problem formulation

Consider a multiplayer reach–avoid differential game in the 3D Euclidean space ℝ^3^ with *Np* pursuers and *Ne* evaders. The target region is a plane Π*_tar_*, and the pursuers strive to capture evaders to prevent them from approaching the target plane while the evaders aim to reach it as far as possible. The group of pursuers is denoted by {*P_i_*, *i* = 1, ⋯, *Np*}, and the group of evaders is denoted by {*E_j_*, *j* = 1, ⋯, *Ne*}. The states of *P_i_* and *E_j_* at time *t* are given by the Cartesian coordinates **x***_P_i__*(*t*) = [*x_P_i__*(*t*), *y_P_i__*(*t*), *z_P_i__*(*t*)] ∈ ℝ^3^ and **x***_E_j__*(*t*) = [*x_E_j__*(*t*), *y_E_j__*(*t*), *z_E_j__*(*t*)] ∈ ℝ^3^, respectively. The full state set of the multiplayer differential game is defined as **x**(*t*) ≔ [*x_P_i__*(*t*), *y_P_i__*(*t*), *z_P_i__*(*t*), *x_E_j__*(*t*), *y_E_j__*(*t*), *z_E_j__*(*t*)] ∈ ℝ^3(*Np* + *Ne*)^ for *i* = 1, ⋯, *Np* and *j* = 1, ⋯, *Ne*. The initial state set is denoted as **x**_0_ ≔ **x**(0). The control inputs of *P_i_* and *E_j_* at time *t* are the unit direction vectors of their instantaneous velocities denoted by **u***_P_i__*(*t*) = [*u_P_i__^x^*(*t*), *u_P_i__^y^*(*t*), *u_P_i__^z^*(*t*)] ∈ ℝ^3^ and **u***_E_j__*(*t*) = [*u_E_j__^x^*(*t*), *u_E_j__^y^*(*t*), *u_E_j__^z^*(*t*)] ∈ ℝ^3^, respectively, where ‖**u***_P_i__*(*t*)‖_2_ = 1 and ‖**u***_E_j__*(*t*)‖_2_ = 1. **u***_P_*(*t*) = {**u***_P_i__*(*t*), *i* = 1, ⋯, *Np*} denotes the control set of pursuers. **u***_E_*(*t*) = {**u***_E_j__*(*t*), *j* = 1, ⋯, *Ne*} denotes the control set of evaders. The players have simple motion as Isaacs [11] introduces, i.e., they are permitted to instantaneously adjust their orientations. The dynamics $\overset{\cdot }{x}\left(t\right)=f\left(x\left(t\right),u_{P}\left(t\right),u_{E}\left(t\right)\right)$of the multiplayer differential game are described by the system of 3(*Np* + *Ne*) equations for *t* ≥ 0:

$$\left\{\begin{array}{c} {\overset{\cdot }{x}}_{P_{i}}\left(t\right)=V_{P_{i}}u_{P_{i}}^{x}\left(t\right),{\overset{\cdot }{y}}_{P_{i}}\left(t\right)=V_{P_{i}}u_{P_{i}}^{y}\left(t\right),{\overset{\cdot }{z}}_{P_{i}}\left(t\right)=V_{P_{i}}u_{P_{i}}^{z}\left(t\right)\\ {\overset{\cdot }{x}}_{E_{j}}\left(t\right)=V_{E_{j}}u_{E_{j}}^{x}\left(t\right),{\overset{\cdot }{y}}_{E_{j}}\left(t\right)=V_{E_{j}}u_{E_{j}}^{y}\left(t\right),{\overset{\cdot }{z}}_{E_{j}}\left(t\right)=V_{E_{j}}u_{E_{j}}^{z}\left(t\right) \end{array}\right.$$ (1)

where *V_P_i__* > 0 and *V_E_j__* > 0 are the speeds of *P_i_* and *E_j_*, respectively. For the sake of symbolic brevity, the time *t* will be omitted hereafter.

The point capture is considered in this paper. Without loss of generality, we assume that the target plane Π*_tar_* is delineated by {**x** ∈ ℝ^3^∣*z* = 0}, and all the players located in the region {**x** ∈ ℝ^3^∣*z* > 0} and their initial positions do not coincide. The game terminates with the pursuers winning if the pursuers capture all evaders, and with the evaders winning if any evader reaches Π_*tar*_.

Consistent with current research on the differential game, we also consider players with constant speed. However, the difference lies in our support for heterogeneous players, that is, both the pursuers and evaders can have different speeds. With respect to the speed ratio, in combination with the capture way, there are several cases. For the point capture, if *V_E_j__* > *V_P_i__*, the evader can always escape without being captured; if *V_E_j__* ≤ *V_P_i__*, the capture of the evader by the pursuer is a feasible outcome, which is the focus of most recent work. In the case of the radius capture, the speed ratio will not have a decisive impact on the outcome, and the analysis is relatively simple, which is not within the scope of consideration in this paper, so the following assumption is given.

Assumption 1. The speed ratio of evader *E_j_* and pursuer *P_i_* is assumed to be *V_E_j__*/*V_P_i__* = *w_ij_*, *w_ij_* < 1, for *b_ij_* = 1, ∀ *i* = 1, ⋯, *Np*, ∀ *j* = 1, ⋯, *Ne*.

### Multiplayer differential game

The differential game is usually divided into the game of kind and the game of degree. The game of kind determines who will win given the initial states of players by constructing the barrier, which separates the game space into 2 disjoint parts: pursuers’ winning region $W_{P}$ and evaders’ winning region $W_{P}$. The game of degree gives the optimal strategies for pursuers and evaders in the specified winning region, which is transformed into the Value of the game by maximizing and minimizing the payoff with the property of a continuum of numerical values. In this paper, we focus on the game of degree in the pursuers’ winning region $W_{P}$, where the pursuers are guaranteed to capture all evaders and win the game, to solve the optimal strategies for players. Moreover, the game of kind is also discussed in the “Apollonius Sphere” section.

The information pattern is essential for players in the differential game. The non-anticipative information pattern is a commonly used one where the player has access to all states and the opponent’s current control inputs. Stackelberg game employs this information pattern. However, considering the rationality of the adversarial game, the opponent’s control inputs should be unavailable; thus, the state feedback information pattern is employed in this paper, where players are only privy to the state information, and not the control inputs of their opponents. The objective of this paper is to determine the optimal strategy based on the state feedback information, which is also referred to as the optimal state feedback strategy.

For the multiplayer differential game, we define a binary matching matrix for pursuers and evaders: **B** = [*b_ij_*]_*Np*×*Ne*_, and *b_ij_* = 1 if *P_i_* is assigned to *E_j_*; otherwise, *b_ij_* = 0. Note that one pursuer is only assigned to one evader, one evader is assigned to one pursuer when *Np* = *Ne*, and one evader is assigned to multiple pursuers when *Np* > *Ne*, and the case with *Np* < *Ne* is not considered. The matching matrix does not change once the assignment is determined throughout the game.

In the pursuers’ winning region $W_{P}$, the game terminates when *P_i_* captures *E_j_* for all matching pairs *P_i_* − *E_j_*. Therefore, the terminal set D of pursuers is described by

$$D≔\left\{x|x_{P_{i}}=x_{E_{j}},\forall j=1,\;\cdots \;,\mathrm{Ne},\exists i=1,\cdots ,\mathrm{Np},\text{and}\;b_{\mathrm{ij}}=1\right\}$$ (2)

With respect to the target plane {**x** ∈ ℝ^3^∣*z* = 0}, the purpose of evaders is to make *z*-coordinates as small as possible when captured. The pursuers’ objective is just the opposite. Hence, the terminal payoff function *J* can be constructed using the terminal state, which is of Mayer-type and defined as

$$J\left(u_{P},u_{E};x_{0}\right)=\sum_{j=1}^{\mathrm{Ne}}z_{E_{j}}\left(t_{f}\right)$$ (3)

where *t_f_* is the terminal time of the game. Then, the objective of pursuers and evaders yields the Value of the game *V* described as

$$V\left(x_{0}\right)≔\underset{u_{P}}{\text{max}}\;\underset{u_{E}}{\text{min}}\;J\left(u_{P},u_{E};x_{0}\right)$$ (4)

The generic theorem for the differential game is given as follows, which is a primary cornerstone for subsequent analysis in this paper.

**Theorem 1 (Constant optimal strategy).** For the differential game wherein the players have simple motion described as Eq. 1 and the payoff function is of Mayer-type, all players’ optimal strategies are constant, and their optimal trajectories are straight lines.

**Proof.** Let **λ** = [*λ_P_i__^x^*, *λ_P_i__^y^*, *λ_P_i__^z^*, *λ_E_j__^x^*, *λ_E_j__^y^*, *λ_E_j__^z^*]^T^ ∈ ℝ^3(*Np* + *Ne*)^ be the costate variable, for *i* = 1, ⋯, *Np*,*j* = 1, ⋯, *Ne*. The Hamiltonian of the multiplayer differential game is

$$\begin{array}{c} H=\sum_{i=1}^{\mathrm{Np}}V_{P_{i}}\left(\lambda_{P_{i}}^{x}u_{P_{i}}^{x}+\lambda_{P_{i}}^{y}u_{P_{i}}^{y}+\lambda_{P_{i}}^{z}u_{P_{i}}^{z}\right)+\\ \sum_{j=1}^{\mathrm{Ne}}V_{E_{j}}\left(\lambda_{E_{j}}^{x}u_{E_{j}}^{x}+\lambda_{E_{j}}^{y}u_{E_{j}}^{y}+\lambda_{E_{j}}^{z}u_{E_{j}}^{z}\right) \end{array}$$ (5)

The optimal strategies of all the players in terms of the costate variables can be obtained from $\underset{u_{E}}{\text{min}}\;\underset{u_{P}}{\text{max}}\;H$, and they are characterized by

$$\left\{ \begin{array}{l} u_{P_{i}}^{x*}=\frac{\lambda_{P_{i}}^{x}}{\sqrt{{\left(\lambda_{P_{i}}^{x}\right)}^{2}+{\left(\lambda_{P_{i}}^{y}\right)}^{2}+{\left(\lambda_{P_{i}}^{z}\right)}^{2}}},u_{E_{j}}^{x*}=-\frac{\lambda_{E_{j}}^{x}}{\sqrt{{\left(\lambda_{E_{j}}^{x}\right)}^{2}+{\left(\lambda_{E_{j}}^{y}\right)}^{2}+{\left(\lambda_{E_{j}}^{z}\right)}^{2}}}\\ u_{P_{i}}^{y*}=\frac{\lambda_{P_{i}}^{y}}{\sqrt{{\left(\lambda_{P_{i}}^{x}\right)}^{2}+{\left(\lambda_{P_{i}}^{y}\right)}^{2}+{\left(\lambda_{P_{i}}^{z}\right)}^{2}}},u_{E_{j}}^{y*}=-\frac{\lambda_{E_{j}}^{y}}{\sqrt{{\left(\lambda_{E_{j}}^{x}\right)}^{2}+{\left(\lambda_{E_{j}}^{y}\right)}^{2}+{\left(\lambda_{E_{j}}^{z}\right)}^{2}}}\\ u_{P_{i}}^{z*}=\frac{\lambda_{P_{i}}^{z}}{\sqrt{{\left(\lambda_{P_{i}}^{x}\right)}^{2}+{\left(\lambda_{P_{i}}^{y}\right)}^{2}+{\left(\lambda_{P_{i}}^{z}\right)}^{2}}},u_{E_{j}}^{z*}=-\frac{\lambda_{E_{j}}^{z}}{\sqrt{{\left(\lambda_{E_{j}}^{x}\right)}^{2}+{\left(\lambda_{E_{j}}^{y}\right)}^{2}+{\left(\lambda_{E_{j}}^{z}\right)}^{2}}} \end{array}\right.$$ (6)

Because the costate variable dynamics satisfy $\overset{\cdot }{\lambda }=-\frac{\partial H}{\partial x}=0$, **λ** is constant, and thus *u_P_i__*^x*^, *u_P_i__*^y*^, *u_P_i__*^z*^ for *i* = 1, ⋯, *Np* and *u_E_j__*^x*^, *u_E_j__*^y*^, *u_E_j__*^z*^ for *j* = 1, ⋯, *Ne* are constant. Consequently, the optimal trajectories of all the players are straight lines.

## Apollonius Sphere

It is well known that the Apollonius circle serves as a powerful tool for addressing pursuit games in the 2D plane, as initially employed by Isaacs in solving simple pursuit games of kind [11]. We expand it to the Apollonius sphere in 3D space, which allows for the analysis of both the game of kind and the game of degree. This extension forms the essence of deriving optimal strategies for the players. By leveraging the geometric properties of the Apollonius sphere, we not only address the 3D space differential game but also facilitate the determination of numerical solutions.

The Apollonius sphere is the set of points in 3D space that have a specified ratio of distances to 2 fixed points. The specific definition and properties are presented in Lemma 1.

**Lemma 1 (Apollonius sphere).** Given 2 fixed points *M*(*x_m_*, *y_m_*, *z_m_*) and *N*(*x_n_*, *y_n_*, *z_n_*), the set of points *P*(*x_p_*, *y_p_*, *z_p_*) satisfying *MP*/*NP* = *w*(*w* > 0, *w* ≠ 1) is defined as the Apollonius sphere *O* of *M* and *N*. If 0 < *w* < 1, the sphere’s center is $O\left(\frac{x_{m}-w^{2}x_{n}}{1-w^{2}},\frac{y_{m}-w^{2}y_{n}}{1-w^{2}},\frac{z_{m}-w^{2}z_{n}}{1-w^{2}}\right)$ and the sphere’s radius is $R=\frac{w\sqrt{{\left(x_{n}-x_{m}\right)}^{2}+{\left(y_{n}-y_{m}\right)}^{2}+{\left(z_{n}-z_{m}\right)}^{2}}}{1-w^{2}}$, and the points *M*, *N*, and *O* are collinear, and *O* is on the extension of the line segment *NM*, *M* is interior to the sphere, and *N* is exterior to the sphere. If *w* > 1, the sphere’s center is $O\left(\frac{w^{2}x_{n}-x_{m}}{w^{2}-1},\frac{w^{2}y_{n}-y_{m}}{w^{2}-1},\frac{w^{2}z_{n}-z_{m}}{w^{2}-1}\right)$ and the sphere’s radius is $R=\frac{w\sqrt{{\left(x_{n}-x_{m}\right)}^{2}+{\left(y_{n}-y_{m}\right)}^{2}+{\left(z_{n}-z_{m}\right)}^{2}}}{w^{2}-1}$, and the points *M*, *N*, and *O* are collinear, and *O* is on the extension of the line segment *MN*, *N* is interior to the sphere, and *M* is exterior to the sphere.

**Proof.** See the Supplementary Materials.

According to Theorem 1 and Lemma 1, the Apollonius sphere can be used to address the differential game in 3D space, which leads to the following theorem and corollaries.

**Theorem 2 (Capture on the Apollonius sphere).** Consider the pursuer *P_i_* and the evader *E_j_* with constant speed *V_P_i__* and *V_E_j__*, and *V_E_j__*/*V_P_i__* = *w_ij_*, *w_ij_* < 1. When they play with the optimal strategies, that is, in a straight line, the pursuer is guaranteed to capture the evader on their Apollonius sphere.

**Fig. 1.**
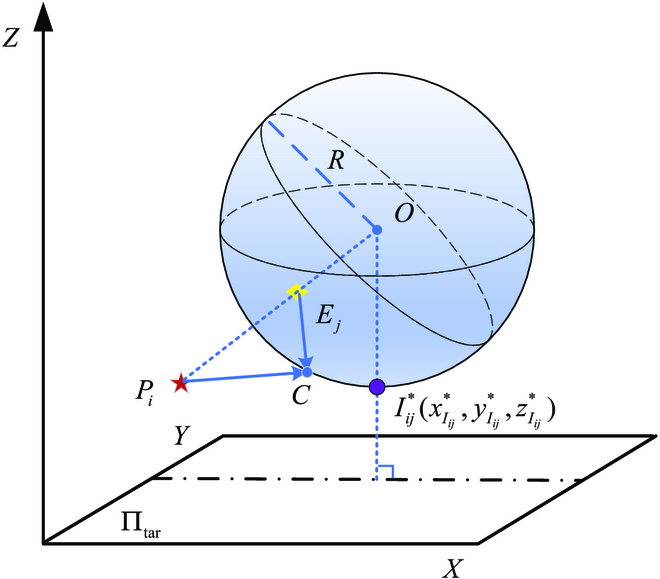
Apollonius sphere and the optimal interception point.

**Proof.** Given the positions of the pursuer *P_i_* and evader *E_j_*, *V_E_j__*/*V_P_i__* = *w_ij_*, *w_ij_* < 1, the Apollonius sphere *O* of *P_i_* and *E_j_* is shown in Fig. 1, where the evader *E_j_* is inside the Apollonius sphere, which implies that the evader *E_j_* is inside the Apollonius sphere, which implies that the evader *E_j_* will pass through the Apollonius sphere no matter which direction it goes. Suppose the point *C* is a random point on the Apollonius sphere, according to the properties of the Apollonius sphere, *E_j_C*/*P_i_C* = *w_ij_*. The pursuer and the evader play with the optimal strategy. Suppose that the point *C* is their target point, then the pursuer and the evader move in a straight line toward point *C* with speed *V_P_i__* and *V_E_j__*, respectively. In Δ*P_i_E_j_C*, the following equation holds

$$\frac{E_{j}C}{P_{i}C}=\frac{V_{E_{j}}t_{E_{j}C}}{V_{P_{i}}t_{P_{i}C}}=w_{\mathrm{ij}}$$ (7)

where *t_E_j_C_* and *t_P_i_C_* are the time taken by the evader to move from point *E_j_* to point *C* and the pursuer to move from point *P_i_* to point *C*, at a constant speed in a straight line, respectively.

Thus, *t_E_j_C_* = *t_P_i_C_*, which means that the pursuer *P_i_* will capture the evader *E_j_* at the point *C* on the Apollonius sphere.

**Corollary 1 (Optimal interception point).** Consider the differential game described in Eqs. 1 to 4, $x\in W_{P}$. Given the initial positions of the pursuer *P* and the evader *E*, the optimal interception point I^*^ for *P* capturing *E* is the point on the Apollonius sphere of *P* and *E* closest to the target plane Π*_tar_*. For the pursuer *P_i_* and the evader *E_j_*, the optimal interception point $I_{ij}^{*}(x_{I_{ij}}^{*},y_{I_{ij}}^{*},z_{I_{ij}}^{*})$ is explicitly given by

$$\left\{\begin{array}{c} x_{I_{\mathrm{ij}}}^{*}=\frac{x_{E_{j}}-w_{\mathrm{ij}}^{2}x_{P_{i}}}{1-w_{\mathrm{ij}}^{2}}\\ y_{I_{\mathrm{ij}}}^{*}=\frac{y_{E_{j}}-w_{\mathrm{ij}}^{2}y_{P_{i}}}{1-w_{\mathrm{ij}}^{2}}\\ z_{I_{\mathrm{ij}}}^{*}=\frac{z_{E_{j}}-w_{\mathrm{ij}}^{2}z_{P_{i}}-w_{\mathrm{ij}}\sqrt{{\left(x_{P_{i}}-x_{E_{j}}\right)}^{2}+{\left(y_{P_{i}}-y_{E_{j}}\right)}^{2}+{\left(z_{P_{i}}-z_{E_{j}}\right)}^{2}}}{1-w_{\mathrm{ij}}^{2}} \end{array}\right.$$ (8)

**Proof.** This corollary follows from Theorems 1 and 2 and Lemma 1. The optimal interception point is shown in Fig. 1.

The region inside the Apollonius sphere of *P* and *E* is the dominance region of *E*, where *E* will not be captured regardless of the pursuer’s strategies. Thus, the winning condition of the game can be determined based on the geometric relationship between the dominance region and the target plane, which is summarized in the following corollary.

**Corollary 2 (Game of kind).** Given the initial positions of the pursuer *P* and the evader *E*, the winning condition is as follows: The pursuer wins if the Apollonius sphere of *P* and *E* has no intersection with the target plane. The game is tied if the Apollonius sphere of *P* and *E* is tangent to the target plane. The evader wins if the Apollonius sphere of *P* and *E* intersects the target plane.

**Proof.** This corollary is easily derived from Theorems 1 and 2 and Corollary 1.

## Two Pursuers Two Evaders 3D Reach–Avoid Differential Game

To facilitate understanding and make a specific presentation explicitly, this section addresses a specific scenario of the multiplayer reach–avoid game: 2 pursuers versus 2 evaders. The state set of the 2 pursuers versus 2 evaders 3D reach–avoid game is **x** ≔ [*x*_*P*_1__, *y*_*P*_1__, *z*_*P*_1__, *x*_*P*_2__, *y*_*P*_2__, *z*_*P*_2__, *x*_*E*_1__, *y*_*E*_1__, *z*_*E*_1__, *x*_*E*_2__, *y*_*E*_2__, *z*_*E*_2__] ∈ ℝ^12^. In the pursuer’s winning region, on the one hand, the pursuers aim to capture the evaders as far away from the target plane as possible to achieve the purpose of making the target plane safest; on the other hand, although the evaders cannot guarantee to reach the target plane, they attempt to be as close to the target plane as possible when they are captured. Therefore, with the constant optimal strategies, the optimal interception point on the corresponding Apollonius sphere is their common aimpoint. According to Theorem 1 and Corollary 1, define

$$\left\{\begin{array}{c} f_{1}\left(x\right)=z_{I_{11}}^{*}+z_{I_{22}}^{*}\\ f_{2}\left(x\right)=z_{I_{12}}^{*}+z_{I_{21}}^{*} \end{array}\right.$$ (9)

For the 2 pursuers versus 2 evaders reach–avoid differential game in 3D, the following theorem provides the optimal assignment and the optimal strategies for 2 pursuers and 2 evaders.

**Theorem 3 (Optimal strategies).** Consider the 3D multiplayer differential game described in Eqs. 1 to 4 with 2 pursuers and 2 evaders under Assumption 1, where $x\in W_{P}$. If *f*_1_(**x**) > *f*_2_(**x**), the optimal assignment is *b*_11_ = *b*_22_ = 1, the Value of the game *V*(**x**) = *f*_1_(**x**), and the optimal strategies of all players are given by Eq. 10. *f*_1_(**x**) < *f*_2_(**x**), the optimal assignment is *b*_12_ = *b*_21_ = 1, *V*(**x**) = *f*_2_(**x**), and the optimal strategies of all players are given by Eq. 11. The Value of the game is continuously differentiable on the nonsingular surface and satisfies the HJI equation.

$$\left\{\begin{array}{c} u_{P_{1}}^{x*}=\frac{x_{I_{11}}^{*}-x_{P_{1}}}{d_{I_{11}^{*}P_{1}}},u_{P_{2}}^{x*}=\frac{x_{I_{22}}^{*}-x_{P_{2}}}{d_{I_{22}^{*}P_{2}}},u_{E_{1}}^{x*}=\frac{x_{I_{11}}^{*}-x_{E_{1}}}{d_{I_{11}^{*}E_{1}}},u_{E_{2}}^{x*}=\frac{x_{I_{22}}^{*}-x_{E_{2}}}{d_{I_{22}^{*}E_{2}}}\\ u_{P_{1}}^{y*}=\frac{y_{I_{11}}^{*}-y_{P_{1}}}{d_{I_{11}^{*}P_{1}}},u_{P_{2}}^{y*}=\frac{y_{I_{22}}^{*}-y_{P_{2}}}{d_{I_{22}^{*}P_{2}}},u_{E_{1}}^{y*}=\frac{y_{I_{11}}^{*}-y_{E_{1}}}{d_{I_{11}^{*}E_{1}}},u_{E_{2}}^{y*}=\frac{y_{I_{22}}^{*}-y_{E_{2}}}{d_{I_{22}^{*}E_{2}}}\\ u_{P_{1}}^{z*}=\frac{z_{I_{11}}^{*}-z_{P_{1}}}{d_{I_{11}^{*}P_{1}}},u_{P_{2}}^{z*}=\frac{z_{I_{22}}^{*}-z_{P_{2}}}{d_{I_{22}^{*}P_{2}}},u_{E_{1}}^{z*}=\frac{z_{I_{11}}^{*}-z_{E_{1}}}{d_{I_{11}^{*}E_{1}}},u_{E_{2}}^{z*}=\frac{z_{I_{22}}^{*}-z_{E_{2}}}{d_{I_{22}^{*}E_{2}}} \end{array}\right.$$ (10)

where

$$\begin{array}{l} d_{I_{11}^{*}P_{1}}=\sqrt{{\left(x_{I_{11}}^{*}-x_{P_{1}}\right)}^{2}+{\left(y_{I_{11}}^{*}-y_{P_{1}}\right)}^{2}+{\left(z_{I_{11}}^{*}-z_{P_{1}}\right)}^{2}},\\ d_{I_{22}^{*}P_{2}}=\sqrt{{\left(x_{I_{22}}^{*}-x_{P_{2}}\right)}^{2}+{\left(y_{I_{22}}^{*}-y_{P_{2}}\right)}^{2}+{\left(z_{I_{22}}^{*}-z_{P_{2}}\right)}^{2}},\\ d_{I_{11}^{*}E_{1}}=\sqrt{{\left(x_{I_{11}}^{*}-x_{E_{1}}\right)}^{2}+{\left(y_{I_{11}}^{*}-y_{E_{1}}\right)}^{2}+{\left(z_{I_{11}}^{*}-z_{E_{1}}\right)}^{2}},\\ d_{I_{22}^{*}E_{2}}=\sqrt{{\left(x_{I_{22}}^{*}-x_{E_{2}}\right)}^{2}+{\left(y_{I_{22}}^{*}-y_{E_{2}}\right)}^{2}+{\left(z_{I_{22}}^{*}-z_{E_{2}}\right)}^{2}} \end{array},$$

$$\left\{\begin{array}{c} u_{P_{1}}^{x*}=\frac{x_{I_{12}}^{*}-x_{P_{1}}}{d_{I_{12}^{*}P_{1}}},u_{P_{2}}^{x*}=\frac{x_{I_{21}}^{*}-x_{P_{2}}}{d_{I_{21}^{*}P_{2}}},u_{E_{1}}^{x*}=\frac{x_{I_{21}}^{*}-x_{E_{1}}}{d_{I_{21}^{*}E_{1}}},u_{E_{2}}^{x*}=\frac{x_{I_{12}}^{*}-x_{E_{2}}}{d_{I_{12}^{*}E_{2}}}\\ u_{P_{1}}^{y*}=\frac{y_{I_{12}}^{*}-y_{P_{1}}}{d_{I_{12}^{*}P_{1}}},u_{P_{2}}^{y*}=\frac{y_{I_{21}}^{*}-y_{P_{2}}}{d_{I_{21}^{*}P_{2}}},u_{E_{1}}^{y*}=\frac{y_{I_{21}}^{*}-y_{E_{1}}}{d_{I_{21}^{*}E_{1}}},u_{E_{2}}^{y*}=\frac{y_{I_{12}}^{*}-y_{E_{2}}}{d_{I_{12}^{*}E_{2}}}\\ u_{P_{1}}^{z*}=\frac{z_{I_{12}}^{*}-z_{P_{1}}}{d_{I_{12}^{*}P_{1}}},u_{P_{2}}^{z*}=\frac{z_{I_{21}}^{*}-z_{P_{2}}}{d_{I_{21}^{*}P_{2}}},u_{E_{1}}^{z*}=\frac{z_{I_{21}}^{*}-z_{E_{1}}}{d_{I_{21}^{*}E_{1}}},u_{E_{2}}^{z*}=\frac{z_{I_{12}}^{*}-z_{E_{2}}}{d_{I_{12}^{*}E_{2}}} \end{array}\right.$$ (11)

where

$$\begin{array}{l} d_{I_{12}^{*}P_{1}}=\sqrt{{\left(x_{I_{12}}^{*}-x_{P_{1}}\right)}^{2}+{\left(y_{I_{12}}^{*}-y_{P_{1}}\right)}^{2}+{\left(z_{I_{12}}^{*}-z_{P_{1}}\right)}^{2}},\\ d_{I_{21}^{*}P_{2}}=\sqrt{{\left(x_{I_{21}}^{*}-x_{P_{2}}\right)}^{2}+{\left(y_{I_{21}}^{*}-y_{P_{2}}\right)}^{2}+{\left(z_{I_{21}}^{*}-z_{P_{2}}\right)}^{2}},\\ d_{I_{21}^{*}E_{1}}=\sqrt{{\left(x_{I_{21}}^{*}-x_{E_{1}}\right)}^{2}+{\left(y_{I_{21}}^{*}-y_{E_{1}}\right)}^{2}+{\left(z_{I_{21}}^{*}-z_{E_{1}}\right)}^{2}},\\ d_{I_{12}^{*}E_{2}}=\sqrt{{\left(x_{I_{12}}^{*}-x_{E_{2}}\right)}^{2}+{\left(y_{I_{12}}^{*}-y_{E_{2}}\right)}^{2}+{\left(z_{I_{12}}^{*}-z_{E_{2}}\right)}^{2}} \end{array},$$

where $\left(x_{I_{ij}}^{*},y_{I_{ij}}^{*},z_{I_{ij}}^{*}\right)$ is shown in Eq. 8.

**Proof.** For 2 pursuers versus 2 evaders reach–avoid game, there are 2 possible assignments: *b*_11_ = *b*_22_ = 1, wherein the pursuer *P*_1_ is assigned to *E*_1_, the pursuer *P*_2_ is assigned to *E*_2_, and the payoff function *J* = *f*_1_(**x**). *b*_12_ = *b*_21_ = 1, wherein the pursuer *P*_1_ is assigned to *E*_2_, the pursuer *P*_2_ is assigned to *E*_1_, and the payoff function *J* = *f*_2_(**x**). According to Theorem 2 and Corollary 1, the optimal assignment should make the pursuers achieve maximum payoff. Thus, if *f*_1_(**x**) > *f*_2_(**x**), the optimal assignment is *b*_11_ = *b*_22_ = 1. If *f*_1_(**x**) < *f*_2_(**x**), the optimal assignment is *b*_12_ = *b*_21_ = 1. For the proof of the optimal strategies and the proof that the Value of the game is continuously differentiable on the nonsingular surface and satisfies the HJI equation, see Proof of Theorem 4, which is omitted here for the sake of brevity.

## Multiplayer 3D Reach–Avoid Differential Games via Harris’ Hawks Cooperative Hunting Tactics

### Harris’ Hawks cooperative hunting tactics

Harris’ Hawk (*Parabuteo unicinctus*) is a social medium-large bird of prey that is native to the Americas and found throughout the United States in Arizona, New Mexico, and Texas, usually living in the deserts in the presence of high cacti, holes, and woody shrubs, etc. Raptors often hunt alone, while Harris’ Hawk’s special living environment with the scarcity of food makes for their unique cooperative hunting behaviors. Harris’ Hawks cooperatively hunt in groups of 2 to 6 using different group sizes and cooperative tactics depending on the prey's size, escape patterns, hiding place, and other characteristics. As Bednarz [30] studied with Harris’ Hawks cooperative hunting, hawks conduct team foraging in an organized and strategic manner. Hunts begin early in the morning, when the Harris’ Hawks communicate with each other through chirping, and gather on tall cacti or power poles, and then the hunting team splits into several groups (usually 1 to 3 hawks) and takes turns to fly short distances in search of prey. Some members, responsible for lookout search, often perch on huge trees, cacti, or power poles to find potential prey, and when the prey is once found, other members will be notified quickly to attack the prey. Some members constantly “leapfrog” throughout the group's range to actively seek prey, occasionally regrouping or splitting again, while moving in a general direction, and this “leapfrog” will continue until they can catch prey or it is dark.

**Fig. 2.**
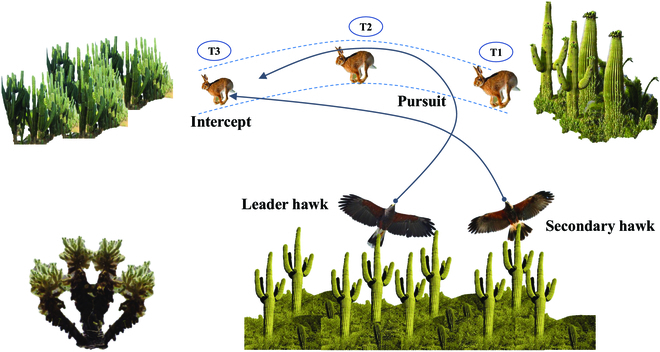
Harris’ Hawk and cooperative tactic. Diagrammatic representation of Harris’ Hawks’ intercept–attack tactic.

Bednarz describes Harris’ Hawk’s most commonly used hunting tactic as a “surprise pounce,” with several hawks flying toward their prey from different directions, and captures can occur instantaneously or may require multiple quick dives to capture prey. The surprise pounce described by Bednarz includes the flush-and-ambush tactic and relay attack tactic; the former is very effective in situations where the prey is hidden by the cover, one hawk flushes into the cover to shock the prey out, and other hawks ambush at the exit of the prey to hunt. The latter refers to the continuous alternation of the leader hawk when chasing prey to ensure that the team’s hunting energy utilization is maximized and the hunting success rate is improved. From a more specific perspective, Dawson [31] summarized 3 Harris’ Hawks cooperative hunting tactics: dual-attack, ascend–attack, and intercept–attack. These tactics can also be classified as a form of “surprise pounce” in certain scenarios. In the dual-attack tactic, 2 hawks fly in the same direction within 1 m of each other, trying to approach the prey at the same time. Two hawks descend together, and the leader hawk tries to touch the prey; if the leader hawk fails, the other will continue to hover above the leader hawk and wait for the opportunity to dive sharply to catch the prey. The ascend–attack tactic is employed when the hawks and prey move in a nearly parallel path. The leader hawk first accelerates and flaps its wings to descend sharply in the direction of the prey, and the other hawk begins to ascend and accelerate. Once the prey escapes, they immediately fly above the prey area and quickly dive to pounce on the prey. When the hawk and the prey move in a nonparallel path, the hunting team will use the intercept–attack tactic. The leader hawk flies directly to the prey and tries to approach and contact the prey, and the second or third hawk flies to the interception path in the area ahead of the prey. The unusual distinction of this tactic is that other hawks do not directly chase the prey but follow the leader hawk to change the flight path and fly to the front of the prey to intercept it. The illustration of the Harris’ Hawks’ intercept–attack tactic is shown in Fig. 2.

Harris’s Hawk cooperative hunting tactics offer many benefits and advantages by improving hunting efficiency, expanding the hunting range, and facilitating social learning. This cooperative behavior enables them to better adapt to the wild environment and increases their chances of successful predation. Inspired by Harris’ Hawk’s cooperative hunting tactics and considering the cooperative problem of the multiplayer reach–avoid game, this paper applies Harris’ Hawk’s intercept–attack tactic to the case of multiple pursuers and one evader, which is further elaborated in the following subsections.

### Optimal strategies for 3D multiplayer reach–avoid differential games with one pursuer one evader pairings

In this subsection, the optimal strategies for 3D multiplayer reach–avoid differential games in the case of *Np* = *Ne* with one pursuer and one evader pairings will be provided. In Harris’ Hawk’s surprise pounce tactic, each hawk in a hunting group selects a potentially captured prey that gives the team the most energy and launches a hunt toward the prey. Based on Corollaries 1 and 2, when dealing with a pairing of one pursuer and one evader, utilizing the Apollonius sphere technique can determine whether the pursuer is capable of capturing the evader. Furthermore, it facilitates the selection of optimal pairings of pursuers and evaders in feasible assignments. Simulating the hawks’ intelligence, the group of pursuers aims to make optimal interception points of each pursuer with respect to the corresponding evader as far away from the target plane as possible, and the group of evaders has the opposite purpose. Define

$$K_{g}\left(x\right)=\sum_{i=1}^{\mathrm{Np}}\sum_{j=1}^{\mathrm{Ne}}b_{\mathrm{ij}}^{g}z_{I_{\mathrm{ij}}}^{*}$$ (12)

where *g* denotes the *g*th assignment. $b_{\mathrm{ij}}^{g}$ = 1 if the pursuer *P_i_* can capture the evader *E_j_* in assignment *g*; otherwise, $b_{\mathrm{ij}}^{g}$ = 0.

Consequently, the following theorem is formulated that gives the solution of the multiplayer differential game in 3D, including both the optimal assignment and the optimal state feedback strategies of all players.

**Theorem 4 (Optimal strategies).** Consider the 3D multiplayer differential game described in Eqs. 1 to 4 with *Np* pursuers and *Ne* evaders (*Np* = *Ne*) under Assumption 1, where $x\in W_{P}$. The Value of the game is $V\left(x\right)={\text{max}}_{g}K_{g}\left(x\right)$, which is continuously differentiable on the nonsingular surface and satisfies the HJI equation. The optimal assignment of the game is $g^{*}=\text{arg}{\text{max}}_{g}K_{g}\left(x\right)$. The optimal strategies of all players for *i* = 1, ⋯, *Np* and *j* = 1, ⋯, *Ne* are given by

$$\left\{\begin{array}{c} u_{P_{i}}^{x*}=\frac{x_{I_{\mathrm{ij}}}^{*}-x_{P_{i}}}{\sqrt{{\left(x_{I_{\mathrm{ij}}}^{*}-x_{P_{i}}\right)}^{2}+{\left(y_{I_{\mathrm{ij}}}^{*}-y_{P_{i}}\right)}^{2}+{\left(z_{I_{\mathrm{ij}}}^{*}-z_{P_{i}}\right)}^{2}}},u_{E_{j}}^{x*}=\frac{x_{I_{\mathrm{ij}}}^{*}-x_{E_{j}}}{\sqrt{{\left(x_{I_{\mathrm{ij}}}^{*}-x_{E_{j}}\right)}^{2}+{\left(y_{I_{\mathrm{ij}}}^{*}-y_{E_{j}}\right)}^{2}+{\left(z_{I_{\mathrm{ij}}}^{*}-z_{E_{j}}\right)}^{2}}}\\ u_{P_{i}}^{y*}=\frac{y_{I_{\mathrm{ij}}}^{*}-y_{P_{i}}}{\sqrt{{\left(x_{I_{\mathrm{ij}}}^{*}-x_{P_{i}}\right)}^{2}+{\left(y_{I_{\mathrm{ij}}}^{*}-y_{P_{i}}\right)}^{2}+{\left(z_{I_{\mathrm{ij}}}^{*}-z_{P_{i}}\right)}^{2}}},u_{E_{j}}^{y*}=\frac{y_{I_{\mathrm{ij}}}^{*}-y_{E_{j}}}{\sqrt{{\left(x_{I_{\mathrm{ij}}}^{*}-x_{E_{j}}\right)}^{2}+{\left(y_{I_{\mathrm{ij}}}^{*}-y_{E_{j}}\right)}^{2}+{\left(z_{I_{\mathrm{ij}}}^{*}-z_{E_{j}}\right)}^{2}}}\\ u_{P_{i}}^{z*}=\frac{z_{I_{\mathrm{ij}}}^{*}-z_{P_{i}}}{\sqrt{{\left(x_{I_{\mathrm{ij}}}^{*}-x_{P_{i}}\right)}^{2}+{\left(y_{I_{\mathrm{ij}}}^{*}-y_{P_{i}}\right)}^{2}+{\left(z_{I_{\mathrm{ij}}}^{*}-z_{P_{i}}\right)}^{2}}},u_{E_{j}}^{z*}=\frac{z_{I_{\mathrm{ij}}}^{*}-z_{E_{j}}}{\sqrt{{\left(x_{I_{\mathrm{ij}}}^{*}-x_{E_{j}}\right)}^{2}+{\left(y_{I_{\mathrm{ij}}}^{*}-y_{E_{j}}\right)}^{2}+{\left(z_{I_{\mathrm{ij}}}^{*}-z_{E_{j}}\right)}^{2}}} \end{array}\right.$$ (13)

for the pairing *P_i_* − *E_j_* subjecting to $b_{\mathrm{ij}}^{g*}$ = 1, where $\left(x_{I_{ij}}^{*},y_{I_{ij}}^{*},z_{I_{ij}}^{*}\right)$ is shown in Eq. 8.

**Proof.** The following proof is based on $b_{\mathrm{ij}}^{g*}$ = 1. First, we prove that the Value function $V\left(x\right)={\text{max}}_{g}K_{g}\left(x\right)$ is continuously differentiable on the nonsingular surface. The partial derivatives of *V*(**x**) with respect to each state variable are as follows.

$$\left\{\begin{array}{c} \frac{\partial V}{\partial x_{P_{i}}}=\frac{-w_{\mathrm{ij}}}{1-w_{\mathrm{ij}}^{2}}\frac{x_{P_{i}}-x_{E_{j}}}{d_{\mathrm{ij}}}\\ \frac{\partial V}{\partial y_{P_{i}}}=\frac{-w_{\mathrm{ij}}}{1-w_{\mathrm{ij}}^{2}}\frac{y_{P_{i}}-y_{E_{j}}}{d_{\mathrm{ij}}}\\ \frac{\partial V}{\partial z_{P_{i}}}=\frac{1}{1-w_{\mathrm{ij}}^{2}}\left(-w_{\mathrm{ij}}^{2}-w_{\mathrm{ij}}\frac{z_{P_{i}}-z_{E_{j}}}{d_{\mathrm{ij}}}\right)=\frac{1}{\left(1-w_{\mathrm{ij}}^{2}\right)d_{\mathrm{ij}}}\left(-w_{\mathrm{ij}}^{2}d_{\mathrm{ij}}-w_{\mathrm{ij}}\left(z_{P_{i}}-z_{E_{j}}\right)\right)\\ \frac{\partial V}{\partial x_{E_{j}}}=\frac{w_{\mathrm{ij}}}{1-w_{\mathrm{ij}}^{2}}\frac{x_{P_{i}}-x_{E_{j}}}{d_{\mathrm{ij}}}\\ \frac{\partial V}{\partial y_{E_{j}}}=\frac{w_{\mathrm{ij}}}{1-w_{\mathrm{ij}}^{2}}\frac{y_{P_{i}}-y_{E_{j}}}{d_{\mathrm{ij}}}\\ \frac{\partial V}{\partial z_{E_{j}}}=\frac{1}{1-w_{\mathrm{ij}}^{2}}\left(1+w_{\mathrm{ij}}\frac{z_{P_{i}}-z_{E_{j}}}{d_{\mathrm{ij}}}\right)=\frac{1}{\left(1-w_{\mathrm{ij}}^{2}\right)d_{\mathrm{ij}}}\left(d_{\mathrm{ij}}+w_{\mathrm{ij}}\left(z_{P_{i}}-z_{E_{j}}\right)\right) \end{array}\right.$$ (14)

where $d_{\mathrm{ij}}=\sqrt{{\left(x_{P_{i}}-x_{E_{j}}\right)}^{2}+{\left(y_{P_{i}}-y_{E_{j}}\right)}^{2}+{\left(z_{P_{i}}-z_{E_{j}}\right)}^{2}}.$

Second, we prove that the Value function $V\left(x\right)={\text{max}}_{g}K_{g}\left(x\right)$ satisfies the HJI equation. The conventional HJI equation is $-\frac{\partial V}{\partial t}=\frac{\partial V}{\partial x}f\left(x,u_{P}^{*},u_{E}^{*}\right)+g\left(t,x,u_{P}^{*},u_{E}^{*}\right)$, but the payoff is of Mayer-type in this problem, so $\frac{\partial V}{\partial t}=0$ and g $g(t,x,u_{p}^{*},u_{E}^{*})=0$ = 0. Therefore, to prove that the Value function satisfies the HJI equation, it is only necessary to prove $\frac{\partial V}{\partial x}f\left(x,u_{P}^{*},u_{E}^{*}\right)=0$.

$$\begin{array}{c} \frac{\partial V}{\partial x}f\left(x,u_{P}^{*},u_{E}^{*}\right)=\\ \sum_{i=1}^{\mathrm{Np}}\sum_{j=1}^{\mathrm{Ne}}V_{P_{i}}\left(\frac{\partial V}{\partial x_{P_{i}}}u_{P_{i}}^{x*}+\frac{\partial V}{\partial y_{P_{i}}}u_{P_{i}}^{y*}+\frac{\partial V}{\partial z_{P_{i}}}u_{P_{i}}^{z*}\right)+\\ V_{E_{j}}\left(\frac{\partial V}{\partial x_{E_{j}}}u_{E_{j}}^{x*}+\frac{\partial V}{\partial y_{E_{j}}}u_{E_{j}}^{y*}+\frac{\partial V}{\partial z_{E_{j}}}u_{E_{j}}^{z*}\right) \end{array}$$ (15)

To obtain it, it is needed to compute the following. According to Eqs. 8 and 14, the following equations hold.

$$\begin{array}{c} \left\{\begin{array}{c} x_{I_{\mathrm{ij}}}^{*}-x_{P_{i}}=\frac{x_{E_{j}}-x_{P_{i}}}{1-w_{\mathrm{ij}}^{2}}=\frac{d_{\mathrm{ij}}}{w_{\mathrm{ij}}}\frac{\partial V}{\partial x_{P_{i}}}\\ y_{I_{\mathrm{ij}}}^{*}-y_{P_{i}}=\frac{y_{E_{j}}-y_{P_{i}}}{1-w_{\mathrm{ij}}^{2}}=\frac{d_{\mathrm{ij}}}{w_{\mathrm{ij}}}\frac{\partial V}{\partial y_{P_{i}}}\\ z_{I_{\mathrm{ij}}}^{*}-z_{P_{i}}=\frac{z_{E_{j}}-z_{P_{i}}-w_{\mathrm{ij}}d_{\mathrm{ij}}}{1-w_{\mathrm{ij}}^{2}}=\frac{d_{\mathrm{ij}}}{w_{\mathrm{ij}}}\frac{\partial V}{\partial z_{P_{i}}}\\ x_{I_{\mathrm{ij}}}^{*}-x_{E_{j}}=\frac{w_{\mathrm{ij}}^{2}\left(x_{E_{j}}-x_{P_{i}}\right)}{1-w_{\mathrm{ij}}^{2}}=-w_{\mathrm{ij}}d_{\mathrm{ij}}\frac{\partial V}{\partial x_{E_{j}}}\\ y_{I_{\mathrm{ij}}}^{*}-y_{E_{j}}=\frac{w_{\mathrm{ij}}^{2}\left(y_{E_{j}}-y_{P_{i}}\right)}{1-w_{\mathrm{ij}}^{2}}=-w_{\mathrm{ij}}d_{\mathrm{ij}}\frac{\partial V}{\partial y_{E_{j}}}\\ z_{I_{\mathrm{ij}}}^{*}-z_{E_{j}}=\frac{w_{\mathrm{ij}}^{2}\left(x_{E_{j}}-x_{P_{i}}\right)-w_{\mathrm{ij}}d_{\mathrm{ij}}}{1-w_{\mathrm{ij}}^{2}}=-w_{\mathrm{ij}}d_{\mathrm{ij}}\frac{\partial V}{\partial z_{E_{j}}} \end{array}\right. \end{array}$$ (16)

Substituting Eq. 16 into Eq. 13 gives

$$\left\{\begin{array}{c} u_{P_{i}}^{x*}=\frac{\frac{\partial V}{\partial x_{P_{i}}}}{\sqrt{{\left(\frac{\partial V}{\partial x_{P_{i}}}\right)}^{2}+{\left(\frac{\partial V}{\partial y_{P_{i}}}\right)}^{2}+{\left(\frac{\partial V}{\partial z_{P_{i}}}\right)}^{2}}}\\ u_{P_{i}}^{y*}=\frac{\frac{\partial V}{\partial y_{P_{i}}}}{\sqrt{{\left(\frac{\partial V}{\partial x_{P_{i}}}\right)}^{2}+{\left(\frac{\partial V}{\partial y_{P_{i}}}\right)}^{2}+{\left(\frac{\partial V}{\partial z_{P_{i}}}\right)}^{2}}}\\ u_{P_{i}}^{z*}=\frac{\frac{\partial V}{\partial z_{P_{i}}}}{\sqrt{{\left(\frac{\partial V}{\partial x_{P_{i}}}\right)}^{2}+{\left(\frac{\partial V}{\partial y_{P_{i}}}\right)}^{2}+{\left(\frac{\partial V}{\partial z_{P_{i}}}\right)}^{2}}} \end{array}\right.$$ (17)

$$\left\{\begin{array}{c} u_{E_{j}}^{x*}=\frac{-\frac{\partial V}{\partial x_{E_{j}}}}{\sqrt{{\left(\frac{\partial V}{\partial x_{E_{j}}}\right)}^{2}+{\left(\frac{\partial V}{\partial y_{E_{j}}}\right)}^{2}+{\left(\frac{\partial V}{\partial z_{E_{j}}}\right)}^{2}}}\\ u_{E_{j}}^{y*}=\frac{-\frac{\partial V}{\partial y_{E_{j}}}}{\sqrt{{\left(\frac{\partial V}{\partial x_{E_{j}}}\right)}^{2}+{\left(\frac{\partial V}{\partial y_{E_{j}}}\right)}^{2}+{\left(\frac{\partial V}{\partial z_{E_{j}}}\right)}^{2}}}\\ u_{E_{j}}^{z*}=\frac{-\frac{\partial V}{\partial z_{E_{j}}}}{\sqrt{{\left(\frac{\partial V}{\partial x_{E_{j}}}\right)}^{2}+{\left(\frac{\partial V}{\partial y_{E_{j}}}\right)}^{2}+{\left(\frac{\partial V}{\partial z_{E_{j}}}\right)}^{2}}} \end{array}\right.$$ (18)

Substituting Eqs. 17 and 18 into Eq. 15 yields

$$\begin{array}{l} \frac{\partial V}{\partial x}f\left(x,u_{P}^{*},u_{E}^{*}\right)=\sum_{i=1}^{\mathrm{Np}}\sum_{j=1}^{\mathrm{Ne}}V_{P_{i}}\frac{{\left(\frac{\partial V}{\partial x_{P_{i}}}\right)}^{2}+{\left(\frac{\partial V}{\partial y_{P_{i}}}\right)}^{2}+{\left(\frac{\partial V}{\partial z_{P_{i}}}\right)}^{2}}{\sqrt{{\left(\frac{\partial V}{\partial x_{P_{i}}}\right)}^{2}+{\left(\frac{\partial V}{\partial y_{P_{i}}}\right)}^{2}+{\left(\frac{\partial V}{\partial z_{P_{i}}}\right)}^{2}}}-V_{E_{j}}\frac{{\left(\frac{\partial V}{\partial x_{E_{j}}}\right)}^{2}+{\left(\frac{\partial V}{\partial y_{E_{j}}}\right)}^{2}+{\left(\frac{\partial V}{\partial z_{E_{j}}}\right)}^{2}}{\sqrt{{\left(\frac{\partial V}{\partial x_{E_{j}}}\right)}^{2}+{\left(\frac{\partial V}{\partial y_{E_{j}}}\right)}^{2}+{\left(\frac{\partial V}{\partial z_{E_{j}}}\right)}^{2}}}\\ \;=\sum_{i=1}^{\mathrm{Np}}\sum_{j=1}^{\mathrm{Ne}}V_{P_{i}}\sqrt{{\left(\frac{\partial V}{\partial x_{P_{i}}}\right)}^{2}+{\left(\frac{\partial V}{\partial y_{P_{i}}}\right)}^{2}+{\left(\frac{\partial V}{\partial z_{P_{i}}}\right)}^{2}}-w_{\mathrm{ij}}V_{P_{i}}\sqrt{{\left(\frac{\partial V}{\partial x_{E_{j}}}\right)}^{2}+{\left(\frac{\partial V}{\partial y_{E_{j}}}\right)}^{2}+{\left(\frac{\partial V}{\partial z_{E_{j}}}\right)}^{2}} \end{array}$$ (19)

where according to Eq. 14 we have that

$$\begin{array}{c} \sqrt{{\left(\frac{\partial V}{\partial x_{P_{i}}}\right)}^{2}+{\left(\frac{\partial V}{\partial y_{P_{i}}}\right)}^{2}+{\left(\frac{\partial V}{\partial z_{P_{i}}}\right)}^{2}}=\\ \frac{w_{\mathrm{ij}}}{\left(1-w_{\mathrm{ij}}^{2}\right)d_{\mathrm{ij}}}\sqrt{d_{\mathrm{ij}}^{2}+w_{\mathrm{ij}}^{2}d_{\mathrm{ij}}^{2}+2w_{\mathrm{ij}}d_{\mathrm{ij}}\left(z_{P_{i}}-z_{E_{j}}\right)} \end{array}$$ (20)

$$\begin{array}{c} \sqrt{{\left(\frac{\partial V}{\partial x_{E_{j}}}\right)}^{2}+{\left(\frac{\partial V}{\partial y_{E_{j}}}\right)}^{2}+{\left(\frac{\partial V}{\partial z_{E_{j}}}\right)}^{2}}=\\ \frac{1}{\left(1-w_{\mathrm{ij}}^{2}\right)d_{\mathrm{ij}}}\sqrt{d_{\mathrm{ij}}^{2}+w_{\mathrm{ij}}^{2}d_{\mathrm{ij}}^{2}+2w_{\mathrm{ij}}d_{\mathrm{ij}}\left(z_{P_{i}}-z_{E_{j}}\right)} \end{array}$$ (21)

Substituting Eqs. 20 and 21 into Eq. 19 gives

$$\begin{array}{l} \frac{\partial V}{\partial x}f\left(x,u_{P}^{*},u_{E}^{*}\right)=\sum_{i=1}^{\mathrm{Np}}\sum_{j=1}^{\mathrm{Ne}}\frac{V_{P_{i}}w_{\mathrm{ij}}}{\left(1-w_{\mathrm{ij}}^{2}\right)d_{\mathrm{ij}}}\sqrt{d_{\mathrm{ij}}^{2}+w_{\mathrm{ij}}^{2}d_{\mathrm{ij}}^{2}+2w_{\mathrm{ij}}d_{\mathrm{ij}}\left(z_{P_{i}}-z_{E_{j}}\right)}\\ \;-\frac{V_{P_{i}}w_{\mathrm{ij}}}{\left(1-w_{\mathrm{ij}}^{2}\right)d_{\mathrm{ij}}}\sqrt{d_{\mathrm{ij}}^{2}+w_{\mathrm{ij}}^{2}d_{\mathrm{ij}}^{2}+2w_{\mathrm{ij}}d_{\mathrm{ij}}\left(z_{P_{i}}-z_{E_{j}}\right)}=0 \end{array}$$ (22)

Thus, the Value function $V\left(x\right)={\text{max}}_{g}K_{g}\left(x\right)$ satisfies the HJI equation.

On the singular surface, there is $K_{g_{1}^{*}}\left(x\right)=K_{g_{2}^{*}}\left(x\right)$, that is, there are 2 or more optimal assignments; hence, the Value of the game is equal on the singular surface and is continuous, but it is not continuously differentiable. For example, suppose *P_i_* is assigned to *E*_*j*_1__ in the optimal assignment $g_{1}^{*}$ , and *P_i_* is assigned to *E*_*j*_2__ in the optimal assignment $g_{2}^{*}$ , then the following inequality holds.

$$\frac{\partial V_{g_{1}^{*}}}{\partial x_{P_{i}}}=\frac{-w_{{\mathrm{ij}}_{1}}}{1-w_{{\mathrm{ij}}_{1}}^{2}}\frac{x_{P_{i}}-x_{E_{j_{1}}}}{d_{{\mathrm{ij}}_{1}}}\neq \frac{\partial V_{g_{2}^{*}}}{\partial x_{P_{i}}}=\frac{-w_{{\mathrm{ij}}_{2}}}{1-w_{{\mathrm{ij}}_{2}}^{2}}\frac{x_{P_{i}}-x_{E_{j_{2}}}}{d_{{\mathrm{ij}}_{2}}}$$ (23)

The remaining corresponding partial derivatives also hold, so it can be proved that the Value of the game on the singular surface is not continuously differentiable.

Remark 1. On the singular surface, the pursuers can select any one among the equally optimal assignments without affecting their payoff. However, to avoid conflicts, one pursuer is given priority and, after determining the optimal assignment, broadcasts the assignment information to other pursuers, thus avoiding the situation where multiple pursuers choose the same evader, and some evaders are missed.

Remark 2. The optimal strategies are the saddle-point strategies of the game, and any player who deviates from the saddle-point strategy will achieve a reduced payoff. The optimal strategies in Eq. 13 are state feedback strategies; thus, players can react in real time to the opponent’s varying strategies. For instance, if the evader employs a non-optimal strategy, the pursuer will continuously recalculate its optimal strategy as given by Eq. 13 and obtain greater benefits. Meanwhile, the evader will not only be captured but also incur higher terminal costs.

### Cooperative pursuit tactic inspired by Harris’ Hawk for multiple pursuers one evader assignments

**Fig. 3.**
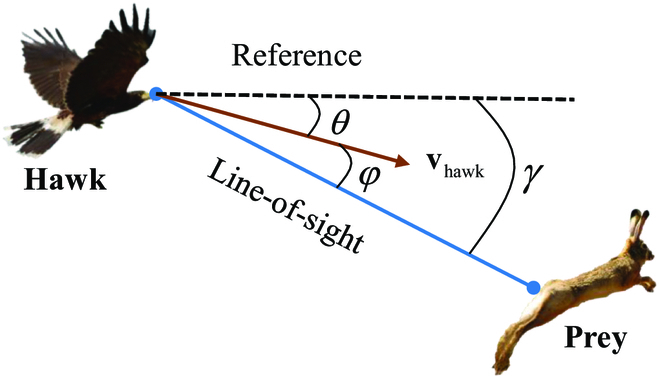
Harris’ Hawk’s pursuit geometry for the prey.

In this subsection, the cooperative tactic for the case of *Np > Ne* with multiple pursuers and one evader assignment is proposed, inspired by Harris’ Hawk’s intercept–attack tactic. In the intercept–attack tactic, the leader hawk is responsible for chasing the prey, and the secondary hawk is designed to intercept the prey on the intended path of the prey. Similarly, in multiple pursuers against one evader game, the pursuers are divided into the leader pursuer and the secondary pursuer. With respect to Harris’ Hawk’s pursuit strategy, the study of Brighton and Taylor [32] demonstrates that Harris’ Hawks use a mixed guidance law to pursue the prey, combining low-gain proportional navigation (PN) with a low-gain pure pursuit (PP). As shown in Fig. 3, the mixed guidance law is given by

$$\overset{\cdot }{\theta }=K_{\mathrm{PN}}\overset{\cdot }{\gamma }-K_{\mathrm{PP}}\varphi$$ (24)

where $\overset{\cdot }{\theta }$ is the turning rate, $\overset{\cdot }{\gamma }$ is the line-of-sight angle rate, and *φ* is the deviation angle between the hawk’s velocity and the line of sight. *K_PN_* is a PN guidance constant, and *K_PP_* is a PP guidance constant. Refer to [32], *K_PN_* = 0.8, *K_PP_* = 1.2.

The Harris’ Hawks’ intercept–attack tactic is used as the cooperative tactic for the pursuers, the leader pursuer adopts Harris’ Hawk’s mixed guidance law to chase the evader, and the secondary pursuer adopts the optimal saddle-point strategy given in Theorem 4 to intercept the evader. With regard to the evaders, their optimal strategies still follow Theorem 4.

**Fig. 4.**
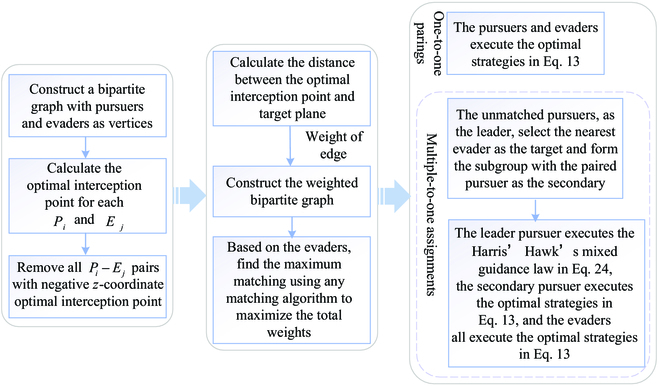
The flowchart of the proposed multiplayer 3D reach–avoid differential games via Harris' Hawks cooperative hunting tactics.

In conclusion, the matching algorithm for multiple pursuers and multiple evaders and the cooperative tactic of the pursuers are presented as follows. Additionally, Fig. 4 displays the flowchart of the proposed multiplayer 3D reach–avoid differential games via Harris' Hawks cooperative hunting tactics.

**Step 1:** Construct a bipartite graph with the pursuers {*P_i_*, *i* = 1, ⋯, *Np*} and evaders {*E_j_*, *j* = 1, ⋯, *Ne*} as 2 node sets, and each player is regarded as a vertex.

**Step 2:** For any *P_i_*, *E_j_*, according to Corollaries 1 and 2, calculate the optimal interception point for *P_i_* to intercept *E_j_*, and an edge is connected to nodes *P_i_* and *E_j_* if *P_i_* can capture *E_j_*. Let the distance between the optimal interception point and the target plane be the weight of this edge.

**Step 3:** Based on the node set of the evaders, find the maximum matching in the bipartite graph using any matching algorithm to maximize the total weights. Hungarian algorithm is employed in this paper.

**Step 4:** The remaining unmatched pursuers, regarded as the leader pursuer, select the nearest evader as the target evader, and the previous pursuer paired with the target evader is regarded as the secondary pursuer. The leader pursuer and the secondary pursuer form a pursuit subgroup, and they attack the target evader adopting the Harris’ Hawks’ intercept–attack cooperative tactic.

Remark 3. The solution obtained for the 3D multiplayer reach–avoid differential game in this paper exhibits excellent scalability with respect to the number of players. As the number of players increases, the solution's performance remains unaffected, and the computation is only slightly increased in the initial multiplayer assignment phase. Furthermore, the proposed method can be implemented in real time, supporting online decision-making in large-scale multiplayer games.

## Simulation Results

This section presents simulation results for 2 representative cases to illustrate the effectiveness of the theoretical outcomes. The simulation analysis is aimed at demonstrating that the optimal state feedback strategy derived in this paper is effective and robust to any unknown strategies of the opponent, that is, regardless of the opponent's strategy, the players can ensure the expected game outcome by implementing the optimal strategies. The optimal strategies for the pursuers will provide a robust solution to capture the evaders, irrespective of the strategies implemented by the evaders (which are unknown to the pursuers). If the pursuer deviates from the saddle-point strategy, the terminal distance between the evader and the target plane will be closer and even the evader may successfully reach the target plane before being captured, thus winning the game. On the other hand, if the evader deviates from the saddle-point strategy, the pursuer will capture it with a smaller cost, i.e., with a larger terminal distance between the evader and the target plane. These purposes highlight the effectiveness of the optimal state feedback strategy, which is the primary result of this paper.

### Case 1: Np = Ne

Considering the case with 2 pursuers and 2 evaders, the initial positions of players are **x**_*P*_1__(0) = [5,10,10], **x**_*P*_2__(0) = [8,25,15], **x**_*E*_1__(0) = [10,10,15], and **x**_*E*_2__(0) = [15,20,18]. The velocities of players are *V*_*P*_1__ = *V*_*P*_2__ = 1 and *V*_*E*_1__ = *V*_*E*_2__ = 0.8. For simplicity, the simulation assumes that the pursuers have identical speeds and the evaders also have identical speeds. However, as long as Assumption 1 is satisfied, both pursuers and evaders are allowed to have different speeds. This same premise applies to the subsequent simulation settings.

**Fig. 5.**
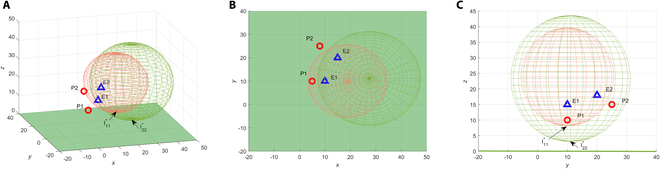
Apollonius spheres and the optimal interception points of *P*_1_*E*_1_ and *P*_2_*E*_2_ parings. (A) 3D view. (B) Top view. (C) Side view.

**Fig. 6.**
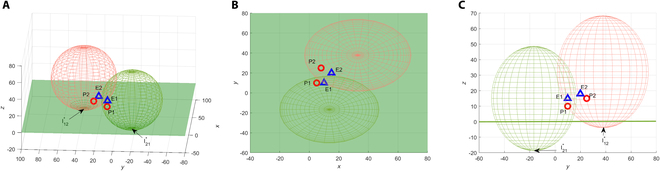
Apollonius spheres and the optimal interception points of *P*_1_*E*_2_ and *P*_2_*E*_1_ parings. (A) 3D view. (B) Top view. (C) Side view.

For 2 pursuers *P*_1_, *P*_2_ and 2 evaders *E*_1_, *E*_2_, the Apollonius sphere of *P*_1_*E*_1_ and the Apollonius sphere of *P*_2_*E*_2_ are shown in Fig. 5, in which the optimal interception point of *P*_1_*E*_1_ is $I_{11}^{*}$ (18.89,10,8.18), and the optimal interception point of *P*_2_*E*_2_ is $I_{22}^{*}$ (27.44,11.11,3.09). Both Apollonius spheres have no intersection with the target plane, indicating that *P*_1_*E*_1_ and *P*_2_*E*_2_ are feasible assignments, and the pursuers are guaranteed to win with the optimal strategies. The Apollonius sphere of *P*_1_*E*_2_ and the Apollonius sphere of *P*_2_*E*_1_ are shown in Fig. 6, in which the optimal interception point of *P*_1_*E*_2_ is $I_{12}^{*}$ (32.78,37.78, − 3.88) and the optimal interception point of *P*_2_*E*_1_ is $I_{21}^{*}$ (13.56, − 16.67, − 18.63), while both Apollonius spheres intersect the target plane, indicating that *P*_1_*E*_2_ and *P*_2_*E*_1_ are invalid assignments, and the evaders can win the game if they adopt the optimal strategies. According to Theorem 3, the optimal assignment of the game is *P*_1_*E*_1_ and *P*_2_*E*_2_. If *P*_1_*E*_2_ and *P*_2_*E*_1_ are also valid assignments, the optimal assignment is the assignment corresponding to the maximum values in $z_{I_{11}}^{*}+z_{I_{22}}^{*}$ and $z_{I_{12}}^{*}+z_{I_{21}}^{*}$ . Three examples are conducted when players adopt optimal or non-optimal strategies to indicate the effectiveness of the proposed solution to the reach–avoid differential game as follows.

***Example 1:*** Pursuers and evaders adopt optimal strategies

**Fig. 7.**
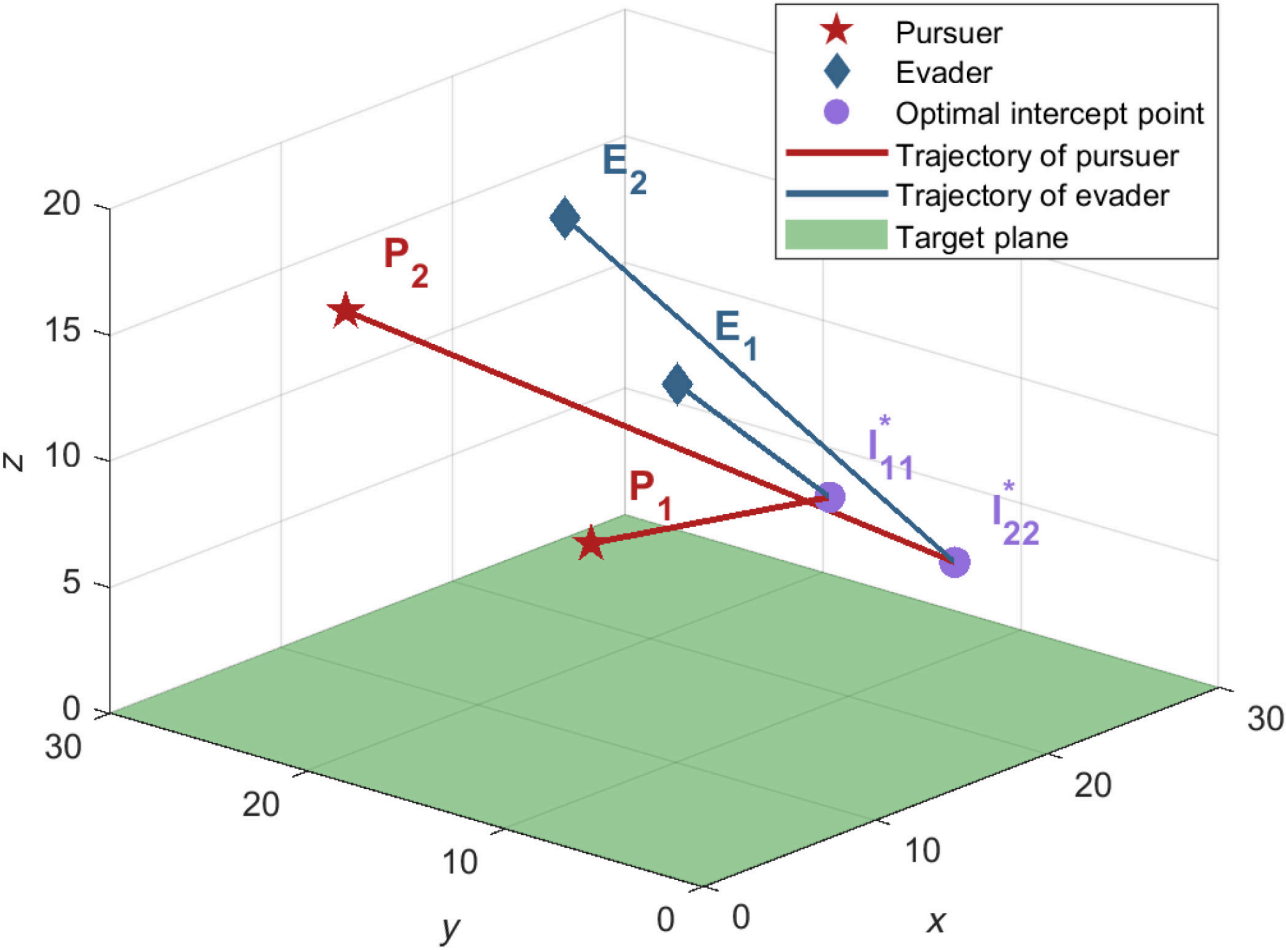
Trajectories with optimal strategies of all players.

According to Theorem 3, the pursuers and the evaders adopt the optimal strategies shown in Eq. 10, the optimal trajectories are shown in Fig. 7, as the theorem states, the optimal interception point is time invariant, and the optimal trajectory is a straight line. The evader *E*_1_ is captured by the pursuer *P*_1_ at the optimal interception point $I_{11}^{*}$ , and the evader *E*_2_ is captured by the pursuer *P*_2_ at the optimal interception point $I_{22}^{*}$ , as expected. The Value of the game is $V=z_{I_{11}}^{*}+z_{I_{22}}^{*}=11.283$ .

***Example 2:*** Pursuers adopt optimal strategies, and evaders adopt non-optimal strategies

In this example, the evaders adopt non-optimal strategies, while the pursuers do not know the evaders’ strategies and execute the optimal strategy and the optimal assignment (*P*_1_*E*_1_ and *P*_2_*E*_2_). As the evaders do not execute the optimal strategy, the time-varying optimal interception point will be generated. The pursuers need to update the optimal strategies by continuously calculating Eq. 10 to react to the evaders' non-optimal strategy. We consider 2 types of non-optimal strategies for the evaders: the first one is a greedy strategy, in which the evaders move directly toward the target plane; the second one is to execute the corresponding optimal strategy based on the non-optimal assignment (*P*_1_*E*_2_ and *P*_2_*E*_1_) with the pursuers.

**Fig. 8.**
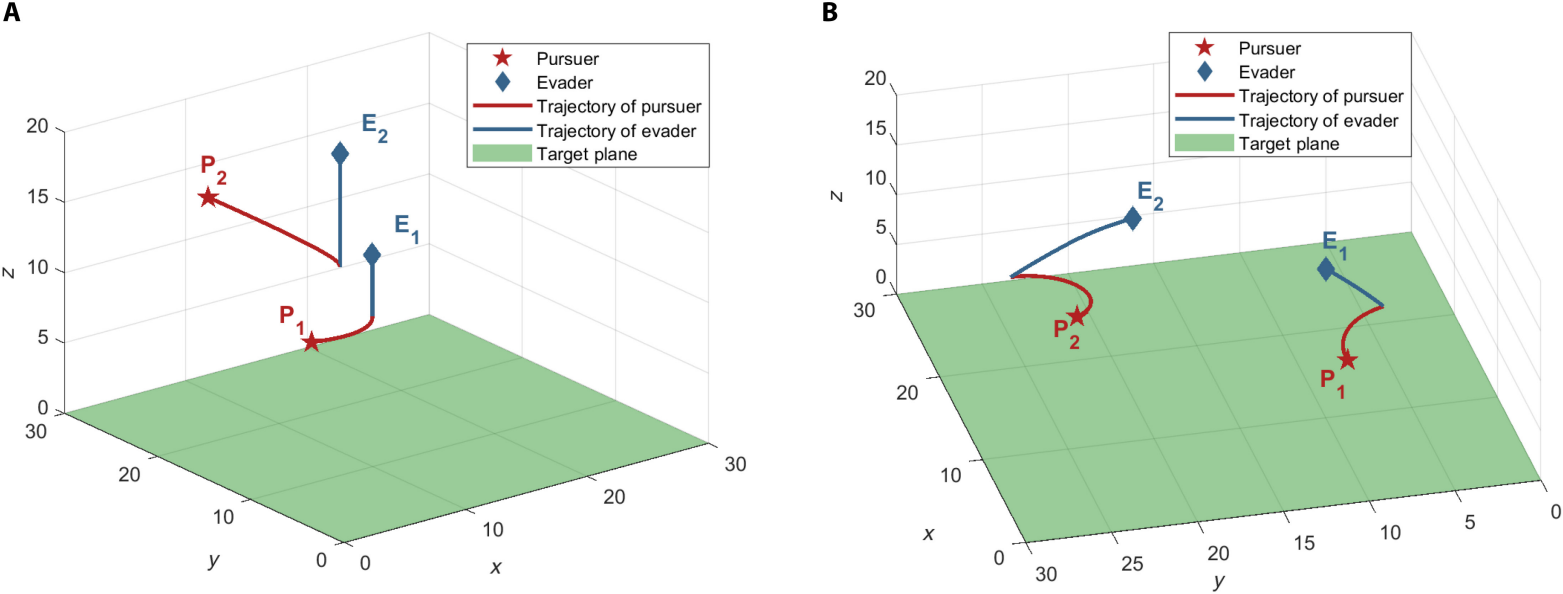
Trajectories with optimal strategies of the pursuers and non-optimal strategies of the evaders. (A) The evaders adopt the greedy strategy. (B) The evaders adopt non-optimal assignment.

The game result in the first case is shown in Fig. 8A, where the evaders are captured faster at a farther distance from the target plane. The terminal payoff (the distance between the evader and the target plane) is $D_{\mathrm{tf}}^{1}$ = 20.6 > *V* = 10.283, indicating that the pursuers win the game with greater profits, while the evader incurs more losses. The game result in the second case is shown in Fig. 8B; the evaders adopt the non-optimal assignment and the optimal strategy, where the evader *E*_1_ incorrectly matches itself with the pursuer *P*_2_ and executes the optimal strategy given by Eq. 11 according to the optimal interception point on the Apollonius sphere of *P*_1_*E*_2_, and the evader *E*_2_ follows the same manner. Finally, the evaders are captured at locations further away from the target plane, resulting in the terminal payoff $D_{\mathrm{tf}}^{2}$ = 21.1744 > *V* = 10.283. These results confirm the robustness of the pursuers’ saddle-point strategy against any strategies of the evaders, as well as the fact that the evaders deviating from the saddle-point strategy led to higher costs.

***Example 3:*** Evaders adopt optimal strategies and pursuers adopt non-optimal strategies

In this example, the evaders adopt optimal strategies, while the pursuers do not. Two cases are considered: In the first case, the pursuer follows optimal assignment (*P*_1_*E*_1_ and *P*_2_*E*_2_) and executes the PP guidance law to pursue its assigned evader; in the second case, the pursuer adopts non-optimal assignment (*P*_1_*E*_2_ and *P*_2_*E*_1_) and executes the optimal strategy given by Eq. 11 on its chosen evader. The evaders do not know the pursuers’ strategies, and they adopt optimal assignment (*P*_1_*E*_1_ and *P*_2_*E*_2_) and the optimal strategies given by Eq. 10. Since the pursuers adopt non-optimal strategies, the optimal interception point is also time varying, and the evaders will constantly update their optimal strategies according to Eq. 10.

**Fig. 9.**
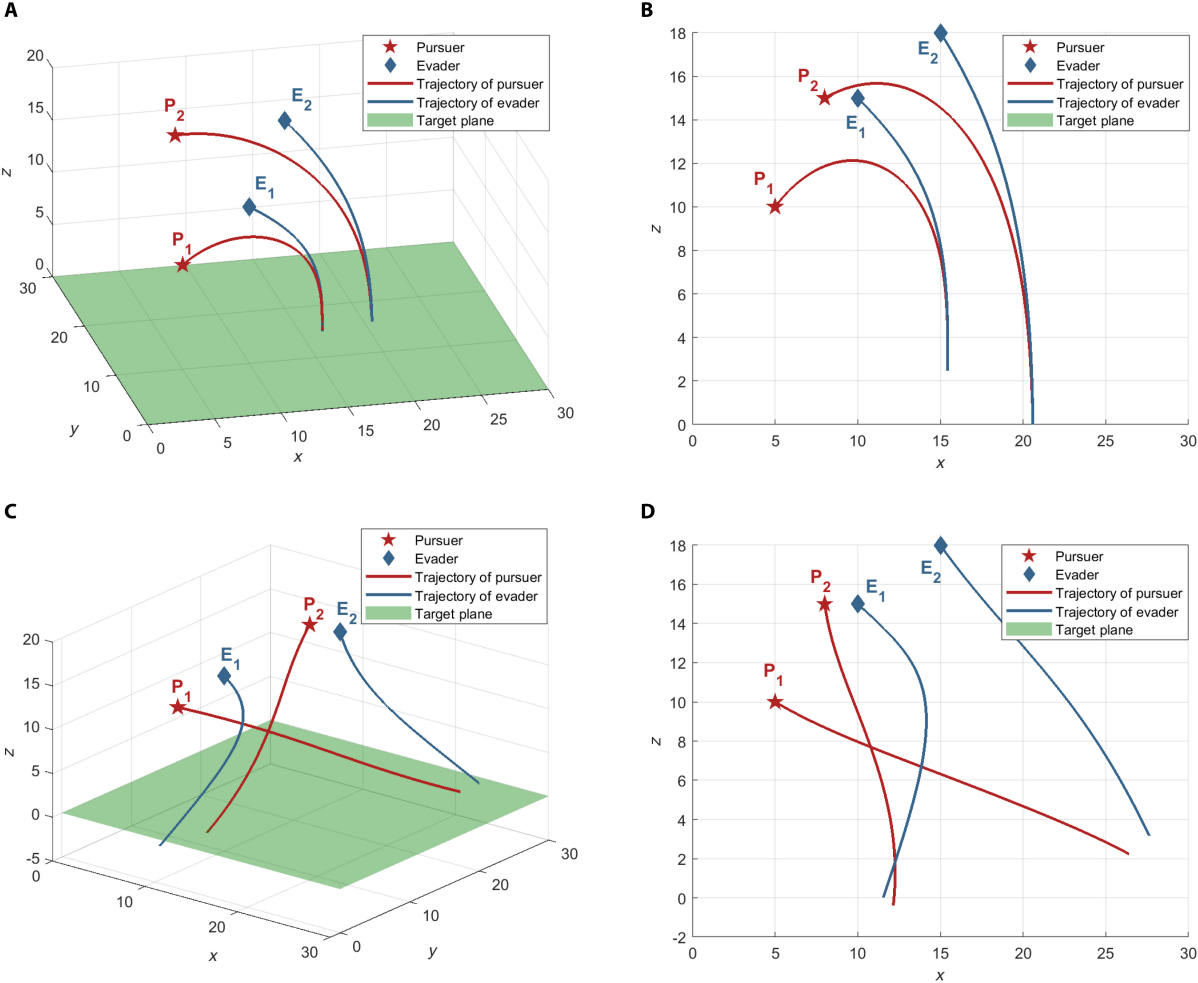
Trajectories with optimal strategies of the evaders and non-optimal strategies of the pursuers. (A) The pursuers adopt optimal assignment and PP guidance law: 3D view. (B) The pursuers adopt optimal assignment and PP guidance law: Side view. (C) The pursuers adopt non-optimal assignment and the optimal strategy: 3D view. (D) The pursuers adopt non-optimal assignment and the optimal strategy: Side view.

The game result in the first case is shown in Fig. 9A and B, although *E*_1_ is captured by *P*_1_ and its terminal distance to the target plane is smaller, *z*_*E*_1__(*t_f_*) = 2.4689. Worse still, *E*_2_ successfully reaches the target plane without being captured by *P*_2_. This shows that even under the optimal assignment, failure to execute the optimal strategy can still degrade the performance of the pursuers. However, due to the non-optimal strategy of the pursuers, the evader implementing the optimal strategy will gain a greater benefit, making it closer to the target plane or even successfully reaching the target plane before being captured. The game result in the second case is shown in Fig. 9C and D, where both *E*_1_ and *E*_2_ are not captured, and *E*_1_ successfully reaches the target plane with the game ends. The terminal distance between *E*_2_ and the target plane is *z*_*E*_2__(*t_f_*) = 3.1659. Under the non-optimal assignment, *P*_1_ selects *E*_2_, and *P*_2_ selects *E*_1_. Although the pursuers adopt the optimal strategy for the assigned evaders, both pursuers failed to capture the corresponding evader. It seemed manifest that the non-optimal strategies result in inferior performance for the pursuers. Only by combining the optimal assignment and optimal strategy can the maximum benefits be achieved.

Based on the findings from the 3 examples, it is evident that the proposed optimal state feedback strategies in this paper exhibit effectiveness and robustness against any unknown opponent strategy, allowing for adaptive responses. Since the optimal state feedback strategy is the saddle-point strategy in the differential game, any deviation from this strategy by a player will inevitably result in inferior outcomes.

### Case 2: Np > Ne

This subsection carries out a simulation to demonstrate the effectiveness of the proposed cooperative tactic and the assignment method in the case of the pursuers outnumbered the evaders (*Np* > *Ne*). Considering the case with 5 pursuers and 3 evaders, the initial positions of players are **x**_*P*_1__(0) = [5,10,10], **x**_*P*_2__(0) = [8,25,15], **x**_*P*_3__(0) = [15,15,16], **x**_*P*_4__(0) = [18,22,14], **x**_*P*_5__(0) = [13,6,12], **x**_*E*_1__(0) = [10,10,15], **x**_*E*_2__(0) = [15,20,18], and **x**_*E*_3__(0) = [10,18,20]. The velocities of players are the same as in Case 1.

**Fig. 10.**
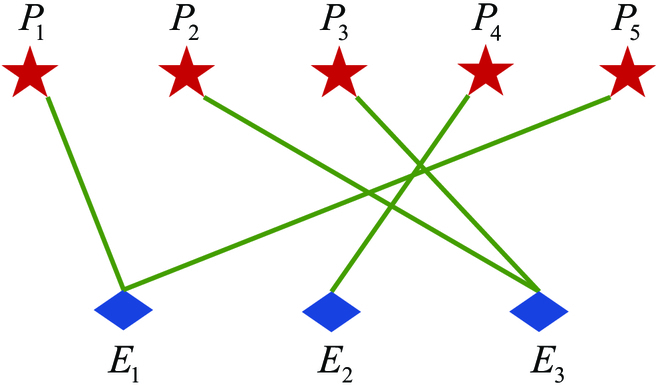
Assignment results for 5 pursuers and 3 evaders.

According to the matching algorithm described in the “Cooperative pursuit tactic inspired by Harris’ Hawk for multiple pursuers one evader assignments” section, the bipartite graph for 5 pursuers and 3 evaders was constructed, the optimal interception point $I_{ii}^{*}$ for each pursuer *P_i_* and evader *E_j_* pairing was calculated, and then the matching weights can be obtained by $z_{I_{\mathrm{ij}}}^{*}$ , shown in Table. Each value in Table represents the distance between the corresponding optimal interception point and the target plane, and the negative value means that the corresponding pursuer cannot capture the evader; hence, the corresponding matching is invalid. Then, the optimal assignment is obtained using the Hungarian algorithm: *P*_1_*E*_1_, *P*_3_*E*_3_, *P*_4_*E*_2_. According to the cooperative tactics presented in the “Cooperative pursuit tactic inspired by Harris’ Hawk for multiple pursuers one evader assignments” section, *P*_1_, *P*_3_, and *P*_4_ serve as the secondary pursuers and adopt the optimal strategies. Furthermore, the remainder pursuers *P*_2_ and *P*_5_ select the nearest evader and serve as the leader pursuer employing Harris’ Hawk’s mixed guidance law, forming the pursuit subgroup of *P*_1_ and *P*_5_, and the pursuit subgroup of *P*_2_ and *P*_3_. The final assignment results are shown in Fig. 10.

**Table.** The matching weights of 5 pursuers to 3 evaders

**Pursuers** **Evaders**	***P*_1_**	***P*_2_**	***P*_3_**	***P*_4_**	***P*_5_**
*E*_1_	8.1754	−18.6283	−2.6476	−15.3485	7.3757
*E*_2_	−3.8846	3.0879	9.5885	13.1441	−5.4718
*E*_3_	7.2273	9.2628	11.3976	6.7326	1.4868

**Fig. 11.**
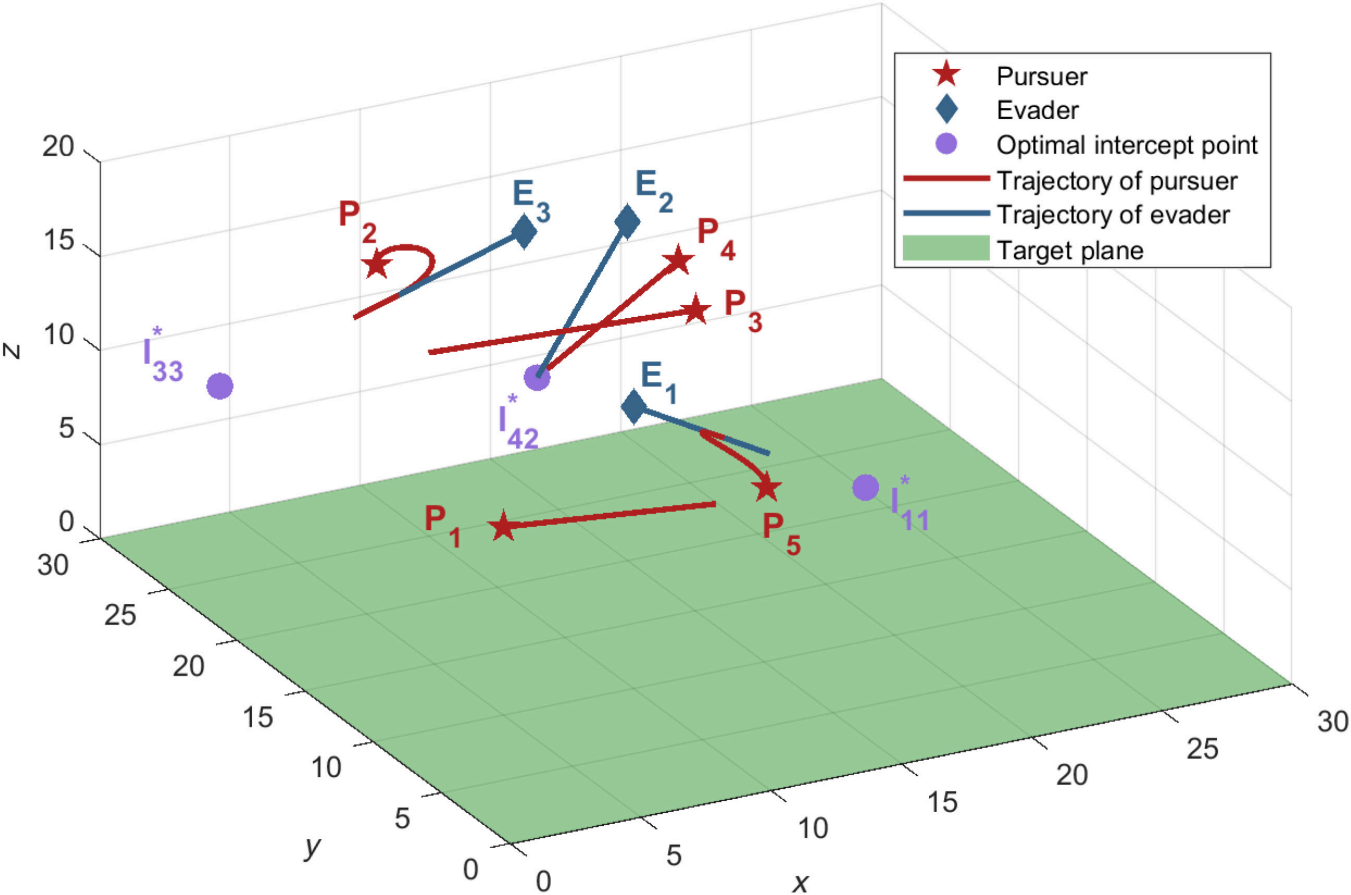
Trajectories for 5 pursuers and 3 evaders.

The trajectories of players are shown in Fig. 11. This multiplayer reach–avoid game terminated with the winning of the pursuers. The whole game is divided into 3 subgames, namely, *P*_1_*P*_5_ − *E*_1_, *P*_2_*P*_3_ − *E*_3_, and *P*_4_ − *E*_2_. For the one-on-one paring *P*_4_ − *E*_2_, the pursuer *P*_4_ and the evader *E*_2_ follow the optimal strategies, and the evader is captured at the optimal interception point $I_{42}^{*}$ . For the multiple pursuers one evader assignment *P*_1_*P*_5_ − *E*_1_, *P*_1_ is the secondary pursuer adopting the optimal strategy and *P*_5_ is the leader pursuer adopting Harris’ Hawk’s mixed guidance law. Finally, the evader *E*_1_ is captured by the leader pursuer *P*_5_ before reaching the optimal interception point $I_{11}^{*}$ , which indicates that the cooperative tactic of *P*_1_ and *P*_5_ is effective and enhances the pursuers group payoff. The scenario with multiple pursuers and one evader assignment *P*_2_*P*_3_ − *E*_3_ resembles the *P*_1_*P*_5_ − *E*_1_ assignment scenario, where the evader *E*_3_ is captured by the leader pursuer *P*_2_ before being intercepted by the secondary pursuer *P*_3_, which demonstrates the effectiveness of Harris’ Hawk’s mixed guidance law.

The comprehensive results of the multiplayer reach–avoid game (*Np* > *Ne*) show the effectiveness of the proposed cooperative tactic and the matching algorithm for multiple pursuers and multiple evaders.

## Conclusions

The multiplayer reach–avoid differential game in 3D space was addressed, where multiple pursuers protect a target plane against multiple evaders. The geometric tool for analyzing 3D differential games, the Apollonius sphere, was utilized. The formulas for solving the center and radius of the Apollonius sphere were given by mathematical proof, and the related properties were proved. It is proved that the capture occurs on the Apollonius sphere under the optimal strategies, and the coordinates of the optimal interception point were given; furthermore, the solution to the game of kind was obtained. Based on the Apollonius sphere, the game of degree in the winning region of the pursuers was considered, and the optimal state feedback strategies and the optimal assignment were presented. The matching algorithm for multiple pursuers and multiple evaders was presented, and the cooperative strategy for multiple pursuers against one evader was designed inspired by Harris’ Hawk’s cooperative hunting tactics. Simulation results demonstrated the effectiveness of the theoretical results of the multiplayer 3D reach–avoid differential game in this paper.

## Data Availability

All data used to support the findings of this study are available from the corresponding author upon request.

## Supplementary Materials

Supplementary 1 Proof of Lemma 1
