# Correction to “Multiplayer Reach–Avoid Differential Games in 3D Space Inspired by Harris’ Hawks’ Cooperative Hunting Tactics”

**DOI:** 10.34133/research.0297

**Published:** 2023-12-28

**Authors:** Wanying Ruan, Haibin Duan, Yongbin Sun, Wanmai Yuan, Jie Xia

**Affiliations:** ^1^ State Key Laboratory of Virtual Reality Technology and Systems, School of Automation Science and Electrical Engineering, Beihang University, Beijing, China.; ^2^ Information Science Academy of CETC, Beijing, China.

## Main Text

In the Research Article “Multiplayer Reach–Avoid Differential Games in 3D Space Inspired by Harris’ Hawks’ Cooperative Hunting Tactics” [1], the publisher made an error. Several equation typos were not corrected during the proofing process.

In the Problem Formulation paragraph beginning with “The point capture is considered in this paper,” the subscript “*tar*” was not italicized. The sentence now reads, “The game terminates with the pursuers winning if the pursuers capture all evaders, and with the evaders winning if any evader reaches π*_tar_*.”

In the Multiplayer differential game paragraph beginning with “For the multiplayer differential game,” all inequalities used “=,” where the second and third should have used “>” and “<,” respectively. The sentence now reads, “Note that one pursuer is only assigned to one evader, one evader is assigned to one pursuer when Np = Ne, and one evader is assigned to multiple pursuers when Np > Ne, and the case with Np < Ne is not considered.”

The maximum value for *H* in Eq. 6 was labeled as u*^P^*. It is now u_P_.

There are two missing square signs in Eq. 6, and the correct equation is as follows:

$$\left\{\begin{array}{l} u_{P_{i}}^{x*}=\frac{\lambda_{P_{i}}^{x}}{\sqrt{{\left(\lambda_{P_{i}}^{x}\right)}^{2}+{\left(\lambda_{P_{i}}^{y}\right)}^{2}+{\left(\lambda_{P_{i}}^{z}\right)}^{2}}},u_{E_{j}}^{x*}=-\frac{\lambda_{E_{j}}^{x}}{\sqrt{{\left(\lambda_{E_{j}}^{x}\right)}^{2}+{\left(\lambda_{E_{j}}^{y}\right)}^{2}+{\left(\lambda_{E_{j}}^{z}\right)}^{2}}}\\ u_{P_{i}}^{y*}=\frac{\lambda_{P_{i}}^{y}}{\sqrt{{\left(\lambda_{P_{i}}^{x}\right)}^{2}+{\left(\lambda_{P_{i}}^{y}\right)}^{2}+{\left(\lambda_{P_{i}}^{z}\right)}^{2}}},u_{E_{j}}^{y*}=-\frac{\lambda_{E_{j}}^{y}}{\sqrt{{\left(\lambda_{E_{j}}^{x}\right)}^{2}+{\left(\lambda_{E_{j}}^{y}\right)}^{2}+{\left(\lambda_{E_{j}}^{z}\right)}^{2}}}\\ u_{P_{i}}^{z*}=\frac{\lambda_{P_{i}}^{z}}{\sqrt{{\left(\lambda_{P_{i}}^{x}\right)}^{2}+{\left(\lambda_{P_{i}}^{y}\right)}^{2}+{\left(\lambda_{P_{i}}^{z}\right)}^{2}}},u_{E_{j}}^{z*}=-\frac{\lambda_{E_{j}}^{z}}{\sqrt{{\left(\lambda_{E_{j}}^{x}\right)}^{2}+{\left(\lambda_{E_{j}}^{y}\right)}^{2}+{\left(\lambda_{E_{j}}^{z}\right)}^{2}}} \end{array}\right.$$

In Eq. 7, factors were mislabeled as $\frac{V_{E_{j}}t_{E_{i}C}}{V_{P_{i}}t_{P_{j}C}}$. The equation should be $\frac{E_{j}C}{P_{i}C}=\frac{V_{E_{j}}t_{E_{j}C}}{V_{P_{i}}t_{P_{i}C}}=w_{\mathrm{ij}}$.

Multiple subscripts were incorrectly labeled as “12” instead of “21” or vice versa in Eq. 11. The correct equation is as follows:

uP1x∗=xI12∗−xP1dI12∗P1,uP2x∗=xI21∗−xP2dI21∗P2,uE1x∗=xI21∗−xE1dI21∗E1,uE2x∗=xI12∗−xE2dI12∗E2uP1y∗=yI12∗−yP1dI12∗P1,uP2y∗=yI21∗−yP2dI21∗P2,uE1y∗=yI21∗−yE1dI21∗E1,uE2y∗=yI12∗−yE2dI12∗E2uP1z∗=zI12∗−zP1dI12∗P1,uP2z∗=zI21∗−zP2dI21∗P2,uE1z∗=zI21∗−zE1dI21∗E1,uE2z∗=zI12∗−zE2dI12∗E2

The same subscript issue extended into the expansions of $d_{I_{21}^{*}E_{1}}$ and $d_{I_{12}^{*}E_{2}}$ following Eq. 11. The correct variables are as follows:

$$d_{I_{21}^{*}E_{1}}=\sqrt{{\left(x_{I_{21}}^{*}-x_{E_{1}}\right)}^{2}+{\left(y_{I_{21}}^{*}-y_{E_{1}}\right)}^{2}+{\left(z_{I_{21}}^{*}-z_{E_{1}}\right)}^{2}},$$

$$d_{I_{12}^{*}E_{2}}=\sqrt{{\left(x_{I_{12}}^{*}-x_{E_{2}}\right)}^{2}+{\left(y_{I_{12}}^{*}-y_{E_{2}}\right)}^{2}+{\left(z_{I_{12}}^{*}-z_{E_{2}}\right)}^{2}}$$

In the Simulation Results-Case 1-Example 2 paragraph beginning with “The game result in the first case is shown in Fig. 8A,” the values of *V* in $D_{\mathrm{tf}}^{1}=20.6>V=10.283$ and $D_{\mathrm{tf}}^{2}=21.1744>V=10.283$ were written incorrectly; they should be the same as $V=z_{I_{11}}^{*}+z_{I_{22}}^{*}=11.283$ in Example 1. The corrected expressions are $D_{\mathrm{tf}}^{1}=20.6>V=11.283$ and $D_{\mathrm{tf}}^{2}=21.1744>V=11.283$.

The authors conclude that these errors did not affect the results, discussion, or conclusion of this paper. The main text and Eqs. 6, 7, and 11 have now been corrected in the PDF and HTML (full text).
