# Supplementary material for: Multiplayer Reach–Avoid Differential Games in 3D Space Inspired by Harris’ Hawks’ Cooperative Hunting Tactics
Source: Research (Wash D C). 2023 Nov 30;6:0246. doi: 10.34133/research.0246 (PMC10768553; doi:10.34133/research.0246)
Supplement: Supplementary 1 — Proof of Lemma 1 [file research.0246.f1.docx]

Multiplayer Reach-Avoid Differential Games in 3D Space Inspired by Harris’ Hawks Cooperative Hunting Tactics

——Supplementary Material——

**Wanying Ruan^1^, Haibin Duan^1,2*^, Yongbin Sun^1^, Wanmai Yuan^3^, Jie Xia^1^**

^1^State Key Laboratory of Virtual Reality Technology and Systems, School of Automation Science and Electrical Engineering, Beihang University, Beijing, China. ^2^Peng Cheng Laboratory, Shenzhen, China. ^3^Information Science Academy of CETC, Beijing, China.

^*^Address correspondence to: hbduan@buaa.edu.cn

This Supplementary Material provides the Proof of Lemma 1.

**Lemma 1 (Apollonius sphere).** Given two fixed points $M\left(x_{m},y_{m},z_{m}\right)$ and $N\left(x_{n},y_{n},z_{n}\right)$, the set of points $P\left(x_{p},y_{p},z_{p}\right)$ satisfying $MP/NP=w\left(w>0,w\neq1\right)$ is defined as the Apollonius sphere $O$ of $M$ and $N$. If $0<w<1$, the sphere’s center is $O\left(\frac{x_{m}-w^{2}x_{n}}{1-w^{2}},\frac{y_{m}-w^{2}y_{n}}{1-w^{2}},\frac{z_{m}-w^{2}z_{n}}{1-w^{2}}\right)$, and the sphere’s radius is $R=\frac{w\sqrt{\left(x_{n}-x_{m}\right)^{2}+\left(y_{n}-y_{m}\right)^{2}+\left(z_{n}-z_{m}\right)^{2}}}{1-w^{2}}$, and the points $M$, $N$, and $O$ are collinear, and $O$ is on the extension of the line segment $NM$, $M$ is interior to the sphere and $N$ exterior; If $w>1$, the sphere’s center is $O\left(\frac{w^{2}x_{n}-x_{m}}{w^{2}-1},\frac{w^{2}y_{n}-y_{m}}{w^{2}-1},\frac{w^{2}z_{n}-z_{m}}{w^{2}-1}\right)$, and the sphere’s radius is $R=\frac{w\sqrt{\left(x_{n}-x_{m}\right)^{2}+\left(y_{n}-y_{m}\right)^{2}+\left(z_{n}-z_{m}\right)^{2}}}{w^{2}-1}$, and the points $M$, $N$, and $O$ are collinear, and $O$ is on the extension of the line segment $MN$, $N$ is interior to the sphere and $M$ exterior.

**Proof.** The following only takes $0<w<1$ as an example to prove, and the same can be proved for $w>1$.

Firstly, we prove that the set of points $P\left(x_{p},y_{p},z_{p}\right)$ is a sphere, meanwhile, the center and radius of the sphere can be obtained.

It follows from $MP/NP=w\left(0<w<1\right)$ that

$\begin{matrix} \frac{\sqrt{\left(x_{p}-x_{m}\right)^{2}+\left(y_{p}-y_{m}\right)^{2}+\left(z_{p}-z_{m}\right)^{2}}}{\sqrt{\left(x_{p}-x_{n}\right)^{2}+\left(y_{p}-y_{n}\right)^{2}+\left(z_{p}-z_{n}\right)^{2}}}=w \\ \Rightarrow\left(x_{p}-x_{m}\right)^{2}+\left(y_{p}-y_{m}\right)^{2}+\left(z_{p}-z_{m}\right)^{2}=w^{2}\left[\left(x_{p}-x_{n}\right)^{2}+\left(y_{p}-y_{n}\right)^{2}+\left(z_{p}-z_{n}\right)^{2}\right] \\ \Rightarrow x_{p}^{2}-2x_{p}x_{m}+x_{m}^{2}+y_{p}^{2}-2y_{p}y_{m}+y_{m}^{2}+z_{p}^{2}-2z_{p}z_{m}+z_{m}^{2} \\ \text{ }=w^{2}\left(x_{p}^{2}-2x_{p}x_{n}+x_{n}^{2}+y_{p}^{2}-2y_{p}y_{n}+y_{n}^{2}+z_{p}^{2}-2z_{p}z_{n}+z_{n}^{2}\right) \\ \Rightarrow\left(1-w^{2}\right)\left(x_{p}^{2}+y_{p}^{2}+z_{p}^{2}\right)-2\left(x_{m}-w^{2}x_{n}\right)x_{p}-2\left(y_{m}-w^{2}y_{n}\right)y_{p} \\ \text{ }-2\left(z_{m}-w^{2}z_{n}\right)z_{p}+x_{m}^{2}+y_{m}^{2}+z_{m}^{2}-w^{2}\left(x_{n}^{2}+y_{n}^{2}+z_{n}^{2}\right)=0 \\ \Rightarrow x_{p}^{2}+y_{p}^{2}+z_{p}^{2}-2\frac{x_{m}-w^{2}x_{n}}{1-w^{2}}x_{p}-2\frac{y_{m}-w^{2}y_{n}}{1-w^{2}}y_{p}-2\frac{z_{m}-w^{2}z_{n}}{1-w^{2}}z_{p} \\ \text{ }+\frac{x_{m}^{2}+y_{m}^{2}+z_{m}^{2}-w^{2}\left(x_{n}^{2}+y_{n}^{2}+z_{n}^{2}\right)}{1-w^{2}}=0 \\ \Rightarrow x_{p}^{2}+y_{p}^{2}+z_{p}^{2}-2\frac{x_{m}-w^{2}x_{n}}{1-w^{2}}x_{p}-2\frac{y_{m}-w^{2}y_{n}}{1-w^{2}}y_{p}-2\frac{z_{m}-w^{2}z_{n}}{1-w^{2}}z_{p} \\ \text{ }+\left(\frac{x_{m}-w^{2}x_{n}}{1-w^{2}}\right)^{2}+\left(\frac{y_{m}-w^{2}y_{n}}{1-w^{2}}\right)^{2}+\left(\frac{z_{m}-w^{2}z_{n}}{1-w^{2}}\right)^{2} \\ \text{ }=\left(\frac{w\sqrt{\left(x_{n}-x_{m}\right)^{2}+\left(y_{n}-y_{m}\right)^{2}+\left(z_{n}-z_{m}\right)^{2}}}{1-w^{2}}\right)^{2} \\ \Rightarrow\left(x_{p}-\frac{x_{m}-w^{2}x_{n}}{1-w^{2}}\right)^{2}+\left(y_{p}-\frac{y_{m}-w^{2}y_{n}}{1-w^{2}}\right)^{2}+\left(z_{p}-\frac{z_{m}-w^{2}z_{n}}{1-w^{2}}\right)^{2} \\ \text{ }=\left(\frac{w\sqrt{\left(x_{n}-x_{m}\right)^{2}+\left(y_{n}-y_{m}\right)^{2}+\left(z_{n}-z_{m}\right)^{2}}}{1-w^{2}}\right)^{2} \end{matrix}$ (1)

Obviously, it is the standard equation of the sphere, and the center coordinates and the radius of the sphere are $O\left(\frac{x_{m}-w^{2}x_{n}}{1-w^{2}},\frac{y_{m}-w^{2}y_{n}}{1-w^{2}},\frac{z_{m}-w^{2}z_{n}}{1-w^{2}}\right)$ and $R=\frac{w\sqrt{\left(x_{n}-x_{m}\right)^{2}+\left(y_{n}-y_{m}\right)^{2}+\left(z_{n}-z_{m}\right)^{2}}}{1-w^{2}}$, respectively.

Secondly, we prove that the points $M$, $N$, and $O$ are collinear. The equation of the straight line determined by $M$ and $N$ is as follows.

$\frac{x-x_{m}}{x_{n}-x_{m}}=\frac{y-y_{m}}{y_{n}-y_{m}}=\frac{z-z_{m}}{z_{n}-z_{m}}$ (2)

Substituting the coordinate of the point $O$ into (2) gives

$\frac{\frac{x_{m}-w^{2}x_{n}}{1-w^{2}}-x_{m}}{x_{n}-x_{m}}=\frac{w^{2}\left(x_{m}-x_{n}\right)}{\left(1-w^{2}\right)\left(x_{n}-x_{m}\right)}=\frac{w^{2}}{w^{2}-1}$ (3)

$\frac{\frac{y_{m}-w^{2}y_{n}}{1-w^{2}}-y_{m}}{y_{n}-y_{m}}=\frac{w^{2}\left(y_{m}-y_{n}\right)}{\left(1-w^{2}\right)\left(y_{n}-y_{m}\right)}=\frac{w^{2}}{w^{2}-1}$ (4)

$\frac{\frac{z_{m}-w^{2}z_{n}}{1-w^{2}}-z_{m}}{z_{n}-z_{m}}=\frac{w^{2}\left(z_{m}-z_{n}\right)}{\left(1-w^{2}\right)\left(z_{n}-z_{m}\right)}=\frac{w^{2}}{w^{2}-1}$ (5)

$\frac{\frac{x_{m}-w^{2}x_{n}}{1-w^{2}}-x_{m}}{x_{n}-x_{m}}=\frac{\frac{y_{m}-w^{2}y_{n}}{1-w^{2}}-y_{m}}{y_{n}-y_{m}}=\frac{\frac{z_{m}-w^{2}z_{n}}{1-w^{2}}-z_{m}}{z_{n}-z_{m}}$ (6)

It can be seen that the point $O$ satisfies the equation of the line $MN$, which proves that the points $M$, $N$, and $O$ are collinear.

Thirdly, we prove that point $O$ is on the extension of the line segment $NM$ from the perspective of the product of the vector.

$\mathop{NM}\limits^{\to} =\left(x_{m}-x_{n},y_{m}-y_{n},z_{m}-z_{n}\right)$ (7)

$\mathop{NO}\limits^{\to} =\left(\frac{x_{m}-w^{2}x_{n}}{1-w^{2}}-x_{n},\frac{y_{m}-w^{2}y_{n}}{1-w^{2}}-y_{n},\frac{z_{m}-w^{2}z_{n}}{1-w^{2}}-z_{n}\right)=\left(\frac{x_{m}-x_{n}}{1-w^{2}},\frac{y_{m}-y_{n}}{1-w^{2}},\frac{z_{m}-z_{n}}{1-w^{2}}\right)$(8)

(9)

Since the points $M$, $N$, and $O$ are collinear, the vector $\mathop{NM}\limits^{\to}$ and $\mathop{NO}\limits^{\to}$ are in the same direction. Besides,

$\left|\mathop{NM}\limits^{\to} \right|=\sqrt{\left(x_{m}-x_{n}\right)^{2}+\left(y_{m}-y_{n}\right)^{2}+\left(z_{m}-z_{n}\right)^{2}}$ (10)

$\left|\mathop{NO}\limits^{\to} \right|=\frac{\sqrt{\left(x_{m}-x_{n}\right)^{2}+\left(y_{m}-y_{n}\right)^{2}+\left(z_{m}-z_{n}\right)^{2}}}{1-w^{2}}$ (11)

$\left|\mathop{NM}\limits^{\to} \right|<\left|\mathop{NO}\limits^{\to} \right|$, due to $0<w<1$. Hence, the point $O$ is on the extension of the line segment $NM$.

Finally, based on the above conclusion it can be easily proved that $M$ is interior to the sphere and $N$ exterior.

$\begin{matrix} MO=\frac{w^{2}\sqrt{\left(x_{m}-x_{n}\right)^{2}+\left(y_{m}-y_{n}\right)^{2}+\left(z_{m}-z_{n}\right)^{2}}}{1-w^{2}} \\ \text{ }<\frac{w\sqrt{\left(x_{n}-x_{m}\right)^{2}+\left(y_{n}-y_{m}\right)^{2}+\left(z_{n}-z_{m}\right)^{2}}}{1-w^{2}}=R \end{matrix}$ (12)

$\begin{matrix} NO=\frac{\sqrt{\left(x_{m}-x_{n}\right)^{2}+\left(y_{m}-y_{n}\right)^{2}+\left(z_{m}-z_{n}\right)^{2}}}{1-w^{2}} \\ \text{ }>\frac{w\sqrt{\left(x_{n}-x_{m}\right)^{2}+\left(y_{n}-y_{m}\right)^{2}+\left(z_{n}-z_{m}\right)^{2}}}{1-w^{2}}=R \end{matrix}$ (13)

Therefore, $M$ is interior to the sphere and $N$ exterior. □
